# Neurobehavioral and Molecular Alterations Following Single and Combined Exposure to Chlorpyrifos and PFHxS in Developing Zebrafish (*Danio rerio*)

**DOI:** 10.3390/toxics14070566

**Published:** 2026-06-27

**Authors:** Eliana Maira Agostini Valle, Amany Sultan, Michelle Puerta, Roomana Shams, Jack Reites, Isaac Konig, Christopher J. Martyniuk

**Affiliations:** 1 Center for Environmental and Human Toxicology, Department of Physiological Sciences, College of Veterinary Medicine, University of Florida, Gainesville, FL 32611, USA; emavalle@unifesp.br (E.M.A.V.); amanysultan2025@gmail.com (A.S.); puertamichelle08@gmail.com (M.P.); shamsroomana4@gmail.com (R.S.); reitesj@ufl.edu (J.R.); isaac.konig@ufrgs.br (I.K.); 2 Instituto de Ciências Ambientais, Químicas e Farmacêuticas, Campus Diadema, Universidade Federal de São Paulo, Diadema 09972-270, São Paulo, Brazil; 3 Animal Health Research Institute, Agriculture Research Center (ARC), Cairo 11381, Egypt; 4 Department of Physiology, University of Veterinary and Animal Sciences, Lahore 51600, Pakistan; 5 Department of Biochemistry, Federal University of Rio Grande do Sul (UFRGS), Porto Alegre 90050-170, Rio Grande do Sul, Brazil; 6 UF Genetics Institute, Interdisciplinary Program in Biomedical Sciences & Neuroscience, University of Florida, Gainesville, FL 32611, USA

**Keywords:** chlorpyrifos, perfluorohexanesulfonic acid, PFAS, pesticides, combined toxicity

## Abstract

Perfluorohexanesulfonic acid (PFHxS) is a per- and polyfluoroalkyl substance (PFAS) frequently detected in aquatic environments, while chlorpyrifos (CPF) is a widely used organophosphate insecticide. Although their individual toxicity is well described, their combined effects remain poorly understood. Here, we evaluated the effects of CPF (0.7 to 700 µg/L), alone or in combination with PFHxS (10 µg/L), in zebrafish embryos. Survival, hatching, malformations, locomotor activity, oxidative stress, apoptosis, and gene expression were assessed after five days of exposure. CPF reduced survival in a concentration-dependent manner, with moderate enhancement under co-exposure, while hatching success was unaffected. Deformities increased with CPF concentration, which remained consistent with PFHxS co-exposure, suggesting toxicity was mediated by CPF. Locomotor activity was largely decreased in a concentration- and phase-dependent manner. No significant changes were observed in ROS levels or apoptosis. Gene expression analysis revealed upregulation of neurotoxicity-related markers (*ache*, *gfap*, *shha*, *syn2a*), particularly at intermediate CPF concentrations and under co-exposure. Oxidative stress-related genes showed differential responses, with *sod1* upregulated and *cat* downregulated only in the combined treatment. Overall, combined exposure did not substantially enhance toxicity compared with CPF alone, suggesting that CPF was the main contributor to the observed effects, whereas PFHxS had limited influence.

## 1. Introduction

Perfluoroalkyl and polyfluoroalkyl substances (PFASs) are compounds that have been used in various products worldwide since the 1940s. Their widespread applications include non-stick coatings, waterproof fabrics, food-contact materials, firefighting foams, and personal care products [1]. These substances are a class of synthetic organic chemicals characterized by at least one fully fluorinated methyl or methylene carbon atom, which confers unique properties [2]. Due to the strength of the carbon–fluorine (C–F) bonds, PFASs exhibit high physical stability, chemical resistance, and high environmental persistence [3]. PFASs are released into aquatic environments from several sources, particularly aqueous film-forming foams (AFFFs), as well as landfill leachates, wastewater effluents, and contaminated soils amended with biosolids. As a result, they are commonly detected as environmental contaminants in both ecosystems and living organisms [4]. These chemicals also stand out for their toxic effects, particularly on aquatic organisms. The main adverse effects include oxidative stress, neurotoxicity, metabolic disruption, reproductive impairment, developmental abnormalities, and immune dysfunction [5,6,7].

**Figure 1:**
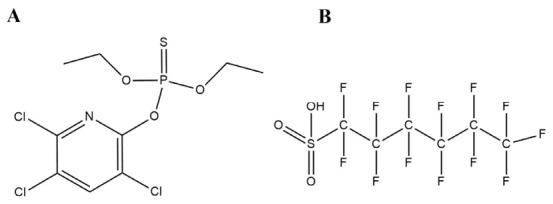
Chemical structures of (**A**) chlorpyrifos (CPF), and (**B**) perfluorohexanesulfonic acid (PFHxS) (**B**).

One perfluoroalkyl substance (PFAS) that has gained increasing attention in recent years due to its frequent detection in various environmental matrices and organisms is perfluorohexanesulfonic acid (PFHxS) [8] (Figure 1). PFHxS is a short-chain PFAS that has been used as a substitute for perfluorooctane sulfonate (PFOS) [9]. A study conducted in a small community in Alaska, investigating the presence of PFASs in residential water and human serum, reported 17 PFAS compounds at elevated concentrations, with PFHxS and PFOS accounting for approximately 70% of the total PFAS burden in those samples [10]. In another study conducted in the Marina catchment, which drains an urbanized area of Singapore, 13 PFAS compounds were detected during both dry and wet weather conditions. PFHxS concentrations ranged from 2.6 to 39.9 ng L^−1^ at different points within the watershed [11]. Furthermore, analysis of 41 surface water samples and edible aquatic organisms collected from Baiyangdian Lake (Hebei Province, China) identified PFOA and PFHxS as the predominant PFASs detected in surface waters, reaching concentrations of 8.40 µg L^−1^ and 1.48 µg L^−1^, respectively [12]. In addition to these studies, PFHxS has been detected in surface soil (median 5.70 µg/kg; maximum 1300 µg/kg) and surface water (median 0.71 µg/L; maximum 815 µg/L) at U.S. Air Force sites impacted by aqueous film-forming foam [13]. Collectively, these data highlight the global distribution and persistence of PFHxS in water and soil, contributing to its bioaccumulation in living organisms, including fish [14] and humans [15]. For instance, in zebrafish larvae, PFHxS exposure (≥2.5 μM) induced developmental abnormalities, including yolk sac edema, delayed brain development, and craniofacial malformations, indicating significant developmental toxicity [16].

Although PFASs are already a significant concern when considered individually, they are not the only contaminants present in the environment. Another major group of pollutants of concern is pesticides [17]. Among various pesticides, chlorpyrifos (CPF) (Figure 1) is a widely used organophosphate insecticide applied to various crops because of its broad-spectrum activity [18,19]. As a result, it has been frequently detected in aquatic environments worldwide. A study evaluating the presence of chlorpyrifos in vegetables and in water samples collected from agricultural fields in Naushahro Feroze Province, Pakistan, at 24 h and 15 days after pesticide application, respectively, showed that a large portion of the pesticide was detected in surface waters, with concentrations ranging from a minimum of 43.46 µg/L to a maximum of 79.7 µg/L [20]. In the Amazon River and in the major nearby urban streams, CPF was detected in 80% of the samples, reaching concentrations up to 0.7 µg/L [21]. CPF and its degradation products were also found in surface water and drinking water from northern Vietnam at an average concentration of 13.4 ng/L [22]. Thus, the widespread occurrence of CPF in water bodies may pose a risk to aquatic organisms.

Exposure to low CPF concentrations is associated with histopathological and behavioral changes, oxidative stress, neurological damage, and genotoxicity [23]. Indeed, adult male zebrafish treated with 2 and 5 μM for 2, 6, and 24 h showed alterations in 50 muscle metabolites, which are linked with oxidative stress, disruption of neurotransmitter metabolism, and muscle exhaustion [24]. Additionally, chronic CPF exposure (3 and 60 μg/L) on adult zebrafish for 90 days impaired reproductive capacity, induced histological damage and transcriptomic changes in the ovary, and disturbed offspring survival and transcriptional profiles in the F1 larvae [25]. In developing zebrafish, CPF exposure at 1 μM was associated with reduced survival and deformities, such as reduced body length and spinal curvature [26]. Some studies have also assessed the impacts of CPF co-exposure on aquatic organisms, including other pesticides [27,28,29], heavy metals [30], and PFASs (perfluorohexanoic acid, PFHxA) [31]. These findings indicate that co-exposure to other contaminants can modulate CPF toxicity, leading to different biological responses. However, the effects of CPF combined with PFHxS are still unknown. In addition, Kienle et al. [30] showed that, compared to developmental and survival parameters, behavior was the most sensitive endpoint for CPF co-exposure, recommending its inclusion to complement traditional toxicity assessments. This reinforces the relevance of evaluating behavioral and neurotoxic endpoints in mixture studies.

Considering these aspects, this study evaluated the effects of chlorpyrifos and PFHxS, individually and in mixtures, on developing zebrafish. Survival rate, hatching success, and the incidence of malformations, apoptosis, reactive oxygen species production, and locomotor activity were assessed. The expression levels of key genes associated with neurotoxicity and oxidative stress were also analyzed. We hypothesized that co-exposure to CPF and PFHxS would alter the toxicity profile observed during single exposures, affecting developmental, behavioral, and molecular endpoints in zebrafish larvae. This study aims to provide data on the combined toxicity of simultaneous exposure to pesticides and PFASs during the early stages of fish development, and to contribute to its risk assessment.

## 2. Materials and Methods

### 2.1. Chemical Compounds

Chlorpyrifos (O,O-diethyl O-3,5,6-trichloro-2-pyridyl phosphorothioate; CPF; CAS 2921-88-2; 98% purity) and perfluorohexanesulfonic acid (1,1,2,2,3,3,4,4,5,5,6,6,6-tridecafluorohexane-1-sulfonic acid; PFHxS; CAS 355-46-4; 74.5% purity) were purchased from Sigma-Aldrich, St. Louis, MO, USA, and LGC Labor GmbH, Augsburg, Germany, respectively. Four CPF stock solutions were prepared in dimethyl sulfoxide (DMSO; CAS 67-68-5; ≥99.9% purity; Sigma-Aldrich, St. Louis, MO, USA) at concentrations of 0.7, 7.0, 70, and 700 μg/mL. Working solutions were obtained by diluting 100 μL of each stock solution into 100 mL of Embryo Rearing Medium (ERM), yielding final CPF concentrations of 0.7, 7.0, 70, and 700 μg/L. For PFHxS, a stock solution was prepared in DMSO and diluted in ERM following the same procedure to obtain a final concentration of 10 μg/L (0.1% DMSO). For mixture treatments, CPF (0.7, 7.0, 70, or 700 μg/L) was combined with PFHxS at a fixed concentration of 10 μg/L. All mixture solutions were prepared using the same dilution procedure, maintaining PFHxS constant at 10 μg/L and DMSO at 0.1%. Negative controls consisted of ERM alone and ERM containing 0.1% DMSO (solvent control). Sterile-filtered ERM was prepared following standard protocols, which can be found in Westerfield [32].

### 2.2. Zebrafish Maintenance and Breeding Procedures

Adult zebrafish (*Danio rerio*; AB × Tübingen strain) were housed in a continuous flow-through aquaculture system (Pentair Aquatic Eco-Systems, Apopka, FL, USA) at the Cancer–Genetics Research Center, University of Florida. Water conditions were maintained within standard laboratory ranges: pH 7.3 ± 1.0, conductivity 600 ± 100 μS·cm^−1^, dissolved oxygen above 80% air saturation, and temperature 28 ± 1 °C. Fish were kept under a 14:10 h light: dark photoperiod and fed a commercial Zeigler Adult Zebrafish Diet to apparent satiation.

For breeding, two males and two females were transferred to spawning tanks on the evening prior to egg collection and separated overnight by a partition. At 08:00 a.m., corresponding to the beginning of the light phase, the divider was removed to initiate spawning. Eggs were collected shortly thereafter, rinsed three times with ERM, and inspected under a stereomicroscope. Unfertilized or morphologically abnormal embryos were excluded. Following collection and selection, embryos were maintained in embryo rearing medium (ERM) at a density of 1 embryo/mL (20 embryos in 20 mL of medium) in glass beakers. Embryos were incubated at 27 ± 1 °C under a 14 h light:10 h dark photoperiod for five days. Dead embryos were removed daily, and 90% of the exposure medium was renewed every 24 h to maintain water quality. Fresh exposure solutions were prepared daily and added following medium renewal. Developmental stages were determined according to morphological criteria and hours post-fertilization as described by Kimmel et al. [33]. All animal procedures were conducted in compliance with protocols approved by the Institutional Animal Care and Use Committee (IACUC) of the University of Florida (Study No. 201708562) and adhered to National Institutes of Health guidelines for the care, use, and humane euthanasia of laboratory animals. Tricaine mesylate (Syncaine, Tricaine-S, Pentair Aquatic Eco-Systems, Inc., Apopka, FL, USA) was used for euthanasia at 250 mg/L buffered with equal parts sodium bicarbonate to a pH of between 7.0 and 7.5.

### 2.3. Exposure Design

Fertilized zebrafish embryos exhibiting normal development were selected at approximately 6 h post-fertilization (hpf) under a stereomicroscope. Embryos were randomly allocated to the following treatment groups (nominal concentrations): ERM (control), 0.1% DMSO (solvent control), CPF at 0.7, 7, 70, and 700 µg/L, PFHxS at 10 µg/L, and CPF/PFHxS mixtures at 0.7/10, 7/10, 70/10, and 700/10 µg/L (*n* = 5 biological replicates, each biological replicate containing 20 embryos/larvae). The CPF concentration range was selected based on previously reported LC_50_ values, spanning environmentally relevant exposures to near-lethal levels to characterize concentration-dependent effects [16,34]. The lower concentrations are environmentally relevant, while the broader range allowed the assessment of concentration-dependent effects. For PFHxS, a single concentration (10 µg/L) was used to evaluate its potential influence on CPF toxicity. This concentration falls within the range reported in areas impacted by aqueous film-forming foams [13].

Four independent experiments were performed using embryos obtained from different breeding pairs. In each experiment, five replicate beakers were assigned to each treatment group, with each beaker containing 20 embryos in 20 mL of exposure solution. Each beaker was considered a biological replicate. Mortality, developmental abnormalities, and hatching rates were monitored daily using an EVOS™ FL Auto Imaging System (Thermo Fisher Scientific, San Diego, CA, USA).

### 2.4. Visual Motor Response

The visual motor response (VMR) assay was conducted according to established procedures [35]. Embryos and larvae remained under continuous exposure to their respective treatments for five days, with 90% of the exposure medium replaced daily as previously described. Experimental groups consisted of ERM (control), 0.1% DMSO (solvent control), CPF (0.7, 7, 70, and 700 µg/L), PFHxS (10 µg/L), and CPF/PFHxS mixtures (0.7/10, 7/10, 70/10, and 700/10 µg/L). On the afternoon of day 5 post-fertilization, two morphologically normal larvae were randomly selected from each replicate beaker and individually transferred to wells of a clear 96-well plate (one larva per well), resulting in 8–10 larvae per treatment per experiment. The experiment was repeated four times (*n* = 18–40 larvae per treatment group). Variation in sample size among groups resulted from the exclusion of larvae with poor tracking quality, which prevented reliable quantification of locomotor activity by the automated tracking software. Each well contained 200 μL of the corresponding exposure solution. Plates were then placed in a DanioVision™ Observation Chamber (Noldus Information Technology, Leesburg, VA, USA) equipped with an infrared camera (25 frames per second) to automatically record locomotor activity. The chamber temperature was maintained at 26 °C.

Locomotor behavior was quantified as total distance traveled. Data were first analyzed separately for each independent experiment. To account for inter-experimental variability, individual locomotor values were normalized to the mean of the corresponding solvent control (0.1% DMSO) within the same phase (light or dark period). This normalization expresses each larva’s activity relative to its internal control group, standardizing data between trials and expressing distance moved as a relative value. Subsequently, normalized data were log-transformed to meet assumptions of normality required for parametric statistical analyses. Thus, all statistical analyses were performed using data that were both normalized and log-transformed. After processing, normalized values from the four independent experiments were pooled, resulting in a final sample size of 18–40 larvae per treatment group.

### 2.5. Reactive Oxygen Species

The assessment of ROS levels was performed following the methodology described by David et al. [36]. Briefly, fertilized embryos were allocated to the respective treatment groups for reactive oxygen species (ROS) determination: ERM (control), 0.1% DMSO (solvent control), CPF at 0.7, 7, and 70 µg/L, PFHxS at 10 µg/L, and CPF/PFHxS combinations at 0.7/10, 7/10, and 70/10 µg/L (*n* = 4 or 5 biological replicates per treatment). Exposure procedures followed the same conditions previously described. After five days of exposure, zebrafish embryos/larvae were euthanized using MS-222, transferred to 1.7 mL microcentrifuge tubes, and homogenized in 200 μL of ice-cold phosphate-buffered saline (PBS). The homogenates were centrifuged at 12,000× *g* for 20 min at 4 °C (LYNX6000, Thermo Scientific, Waltham, MA, USA), and the supernatants were collected for analysis.

For ROS quantification, 20 μL of supernatant was added to a black 96-well fluorescence microplate, followed by 8.3 μL of H_2_-DCFDA solution (1 mg/mL in DMSO) and 200 μL of PBS. The plate was incubated in the dark at 37 ± 1 °C for 30 min. Fluorescence intensity was then measured using a multi-detection microplate reader (New Synergy™, BioTek, Winooski, VT, USA) with excitation at 485 nm and emission at 520 nm. Total protein concentration was determined using the BCA Protein Assay Kit (Thermo Fisher Scientific), and ROS levels were expressed as fluorescence intensity normalized to protein content (μg/mL).

### 2.6. Acridine Orange Staining/Apoptosis Assay

Apoptosis in zebrafish larvae was evaluated using acridine orange staining, as described by Patel et al. [37]. Larvae were maintained for five days under the same experimental conditions previously described, comprising ERM (control), 0.1% DMSO (solvent control), CPF at 0.7, 7, 70, and 700 µg/L, PFHxS at 10 µg/L, and CPF/PFHxS combined treatments at 0.7/10, 7/10, 70/10, and 700/10 µg/L (*n* = 12 larvae per treatment, comprising 4 biological replicates with 3 larvae each). At the end of the exposure period, larvae were rinsed with ERM and subsequently incubated in a 2 μg/mL acridine orange solution (CAS: 65-61-2, Sigma-Aldrich) for 30 min at room temperature in the absence of light. For each biological replicate, three larvae were selected for analysis, totaling 12 larvae per treatment group. After staining, larvae were washed five times with ERM (30 s per wash) to remove excess dye. Imaging was performed using an EVOS FL Auto Imaging System (Thermo Fisher Scientific, USA) equipped with a GFP filter at 10× magnification. Apoptotic cells were identified by the presence of bright green fluorescent signals. Fluorescence intensity was quantified using the histogram tool in ImageJ software, Java 1.8.0.

### 2.7. Real-Time PCR

Zebrafish larvae were exposed for five days to ERM (control), 0.1% DMSO (solvent control), CPF (0.7, 7, 70, and 700 µg/L), PFHxS (10 µg/L), or CPF/PFHxS mixtures (0.7/10, 7/10, 70/10, and 700/10 µg/L) (*n* = 3–5 biological replicates per treatment). Exposures were conducted in Petri dishes (*n* = 5–6 per treatment), each containing 25 embryos. Each Petri dish was considered one biological replicate. At the end of the exposure period, larvae from each dish were pooled and transferred to 1.5 mL microcentrifuge tubes, rapidly frozen in liquid nitrogen, and stored at −80 °C until RNA extraction.

Total RNA was extracted by adding 500 µL of TRIzol^®^ Reagent (Life Technologies, Carlsbad, CA, USA) to each sample, following the manufacturer’s protocol. RNA pellets were resuspended in DNase/RNase-free water and treated with TURBO DNA-free™ (Ambion, Austin, TX, USA) to eliminate residual genomic DNA. Samples were subsequently purified using the RNeasy^®^ Micro Kit (QIAGEN, Hilden, Germany) according to the manufacturer’s instructions and described in the Supplementary Materials.

For cDNA synthesis, approximately 500 ng of purified RNA was reverse-transcribed using the iScript™ cDNA Synthesis Kit (Bio-Rad, Hercules, CA, USA) in a final reaction volume of 15 µL. Reactions were performed in a T100™ Thermal Cycler (Bio-Rad) under the following conditions: 25 °C for 5 min, 42 °C for 30 min, 85 °C for 5 min, and 4 °C for 5 min. Resulting cDNA samples were diluted 1:20 in DNase/RNase-free water prior to quantitative PCR analysis. No-reverse-transcriptase (NRT) controls were prepared using three randomly selected RNA samples processed identically but without the reverse transcriptase enzyme. In addition, two no-template controls (NTCs) were included in the qPCR assays to monitor contamination. Negative controls confirmed effective removal of genomic DNA during column purification and DNase treatment.

Quantitative real-time PCR (qPCR) was carried out using a CFX Connect™ Real-Time PCR Detection System (Bio-Rad) with SsoFast™ EvaGreen^®^ Supermix (Bio-Rad, Hercules, CA, USA), 200–300 nM of each primer, and 3.33 µL of diluted cDNA per reaction. Thermal cycling consisted of an initial enzyme activation step at 95 °C for 30 s, followed by 40 cycles of 95 °C for 5 s and 60 °C for 5 s. A melt curve analysis was performed at the end of amplification (65–95 °C, increasing by 0.5 °C every 5 s) to verify product specificity. All primer pairs amplified a single product, as indicated by a single peak in the melting curve. Primer sequences used in this study are available in Supplemental Table S1 [38,39,40,41,42,43,44].

Gene expression levels were normalized using the combined expression of three reference genes, *rps18*, *β-actin*, and *rpl13a*, which exhibited stability values of M = 0.712 and CV = 1.5577. Relative expression for each target gene was calculated using the Cq method with baseline subtraction in CFX Manager™ software (v3.1). Melt curves and amplification curves for all analyzed genes are provided in the Supplementary Data file to demonstrate assay specificity and amplification quality.

### 2.8. Statistical Analysis

Statistical analyses and graphical representations were performed using GraphPad Prism version 9 (La Jolla, CA, USA). Before the statistical testing, data distribution was evaluated using the Shapiro–Wilk normality test. When necessary, a log_10_ transformation was applied to meet the assumptions of normality. Group comparisons were conducted using one-way analysis of variance (ANOVA), followed by Dunnett’s multiple comparisons test to assess differences relative to the solvent control group (0.1% DMSO). Results are expressed as mean ± standard deviation (S.D.), and statistical significance was established at *p* < 0.05.

## 3. Results

### 3.1. Survival, Hatching Rate, and Morphological Deformities

**Figure 2:**
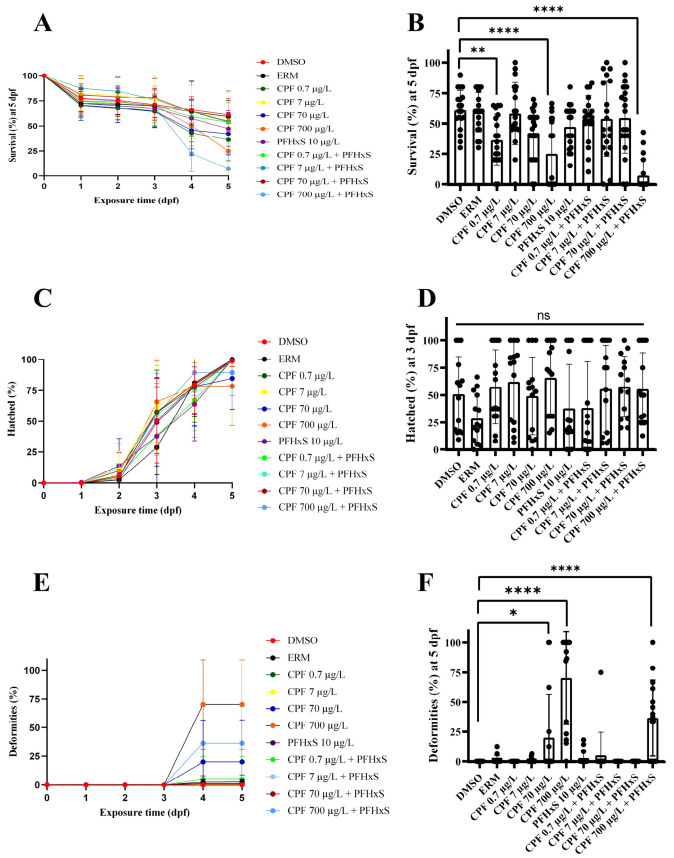
Survival, hatching, and deformities in zebrafish embryos/larvae exposed to chlorpyrifos (CPF) and perfluorohexanesulfonic acid (PFHxS). (**A**) Total survival over 5 days of exposure and (**B**) survival at 5 days post-fertilization (dpf). (**C**) Hatching rate over 5 days and (**D**) cumulative hatching at 3 dpf. (**E**) Incidence of deformities over 5 days and (**F**) cumulative deformities at 5 dpf. Data represent pooled results from four independent experiments, with five beakers per treatment group per experiment. Each biological replicate consisted of 20 embryos. Statistical analysis was performed using one-way ANOVA followed by Dunnett’s post hoc test for comparisons with the solvent control (0.1% DMSO), ns = not significant. * *p* ≤ 0.05, ** *p* ≤ 0.01, **** *p* ≤ 0.001.

CPF and PFHxS exposure effects on survival are shown over time (Figure 2A) and on day 5 (Figure 2B). On the final day, survival at 700 µg/L CPF was approximately 25%, whereas the CPF/PFHxS mixture further reduced survival to ~5%, indicating enhanced toxicity under combined exposure (F_(10, 198)_ = 10.70, *p* < 0.0001). A significant decrease in survival was also observed at 0.7 µg/L CPF. However, this reduction was not detected in the CPF/PFHxS group.

Hatching rates over time (Figure 2C) and at day 5 (Figure 2D) did not differ among treatments (F_(10, 143)_ = 1.51, *p* = 0.14). Only viable embryos were included in the hatching analysis, whereas embryos that did not survive were excluded.

The most frequently observed deformities across treatment groups were spinal lordosis, pericardial edema, and yolk sac edema (Figure S1). The incidence of developmental abnormalities over time is shown in Figure 2E, and cumulative deformities on day 5 are presented in Figure 2F. CPF induced a concentration-dependent increase in malformations, particularly at 70 and 700 µg/L. In the combined treatments, a significant increase in deformities was observed only at 700 µg/L CPF (F_(10, 154)_ = 19.15, *p* < 0.0001). Overall, CPF alone produced concentration-dependent increases in mortality and malformations, and co-exposure to PFHxS resulted in an average of 30% deformed larvae at 5 dpf.

### 3.2. Visual Motor Response

**Figure 3:**
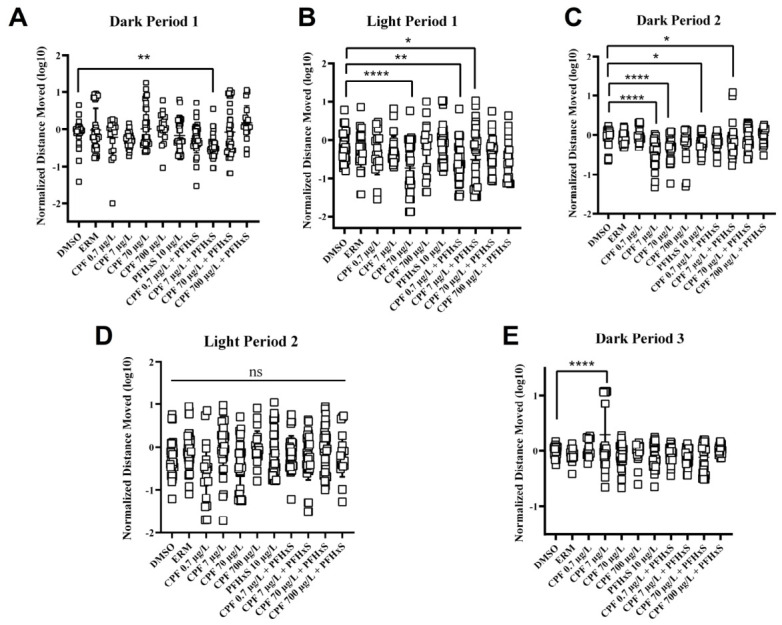
Visual motor response (VMR) of zebrafish larvae continuously exposed to chlorpyrifos (CPF) and perfluorohexanesulfonic acid (PFHxS) for five days. Alternating light–dark cycles were applied in 10-min intervals. (**A**) first dark period, (**B**) first light period, (**C**) second dark period, (**D**) second light period, (**E**) third dark period. Data from four independent experiments were pooled and are presented as a single graph. Statistical analysis was performed using one-way ANOVA followed by Dunnett’s post hoc test for comparisons with the solvent control. ns = not significant (*p* > 0.05); * *p* ≤ 0.05; ** *p* ≤ 0.01; **** *p* ≤ 0.0001. *n* = 18–40 larvae per treatment group.

Locomotor activity was altered in zebrafish larvae following exposure to CPF and PFHxS, either alone or in combination (Figure 3). During the first dark period, exposure to CPF at 7 µg/L combined with PFHxS induced hypoactivity in the larvae (F_(10, 368)_ = 5.07, *p* < 0.0001). Similarly, reduced locomotor activity was observed during the first light cycle for CPF alone at 70 µg/L, as well as in combination with PFHxS (F_(10, 366)_ = 4.76, *p* < 0.0001). However, no differences were detected during the second light cycle compared to the solvent control (F_(10, 364)_ = 3.89, *p* = 0.083). In the second dark period, PFHxS alone induced hypoactivity, as did its combination with CPF at 7 µg/L, and CPF alone at 7 and 70 µg/L (F_(10, 369)_ = 8.96, *p* < 0.0001). In contrast, during the third dark period, exposure to CPF at 7 µg/L increased locomotor activity in the larvae (F_(10, 369)_ = 15.78, *p* < 0.0001). The normalized locomotor activity data for each larva are provided in Supplementary Table S2.

### 3.3. Reactive Oxygen Species

**Figure 4:**
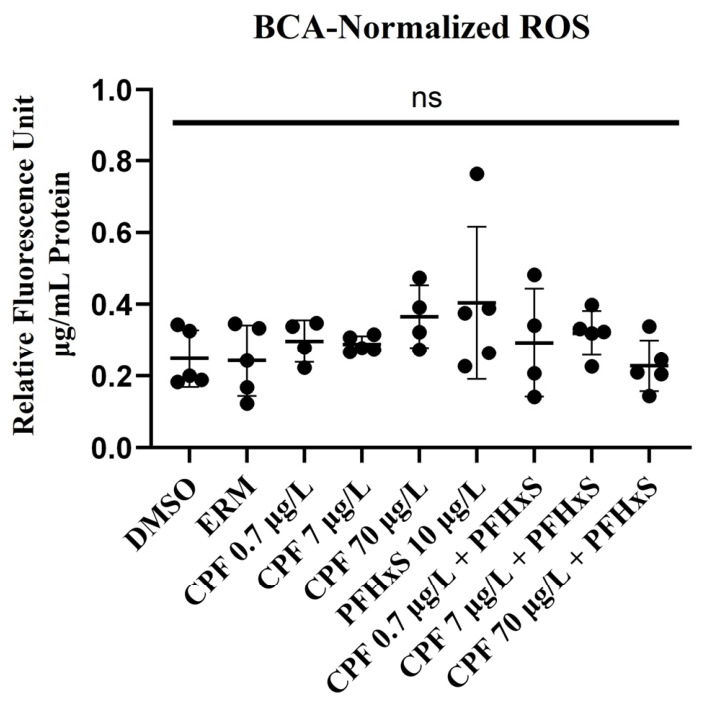
Reactive oxygen species (ROS) levels in 5 dpf zebrafish larvae continuously exposed to chlorpyrifos (CPF) and perfluorohexanesulfonic acid (PFHxS). Treatment groups were: 0.1% DMSO (solvent control), ERM (water control), CPF (0.7, 7.0, or 70 µg/L), PFHxS (10 µg/L), and CPF/PFHxS co-exposure (0.7/10, 7.0/10, or 70/10 µg/L). Fluorescence intensity was quantified as an indicator of intracellular ROS levels. Data was analyzed by one-way ANOVA followed by Dunnett’s post hoc test for comparisons with the solvent control. ns, non-significant (*p* > 0.05). *n* = 4 or 5 biological replicates per treatment.

Reactive oxygen species (ROS) levels were measured in larvae at 5 dpf following exposure to isolated CPF, PFHxS, or their combination (Figure 4). However, no significant differences were detected among treatment groups (F_(8,33)_ = 1.38, *p* = 0.24).

### 3.4. Acridine Orange Staining/Apoptosis Assay

**Figure 5:**
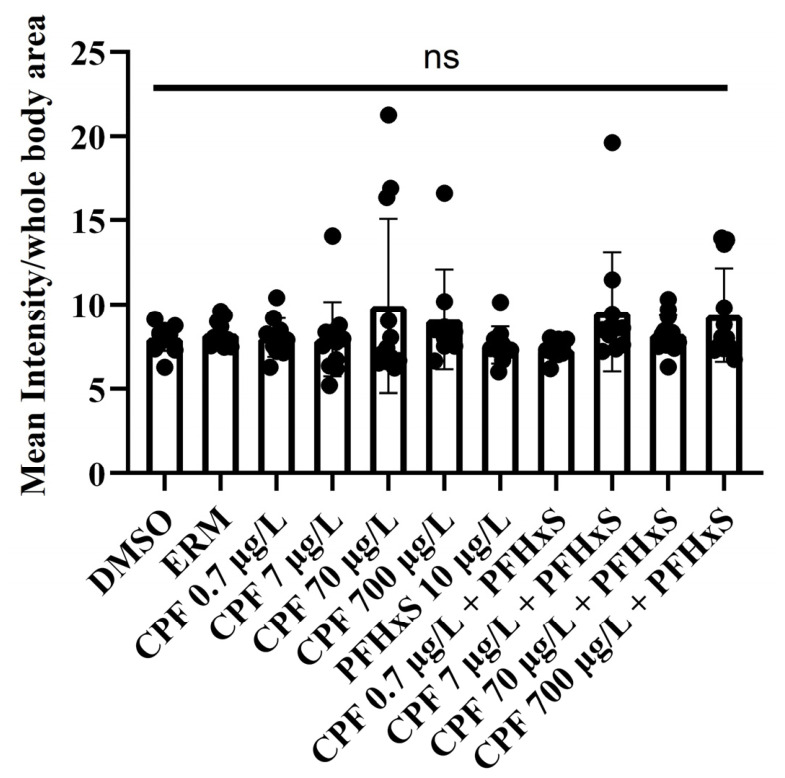
Apoptosis levels assessed by acridine orange staining in zebrafish larvae continuously exposed to chlorpyrifos (CPF) and perfluorohexanesulfonic acid (PFHxS) for five days. Treatment groups were: 0.1% DMSO (solvent control), ERM (water control), CPF (0.7, 7.0, 70, or 700 µg/L), PFHxS (10 µg/L), and CPF/PFHxS co-exposure (0.7/10, 7/10, 70/10, or 700/10 µg/L). Fluorescence intensity was used as an indicator of apoptotic cells. Data were analyzed by one-way ANOVA followed by Dunnett’s post hoc test for comparisons with the solvent control. ns = not significant (*p* > 0.05). *n* = 12 larvae per treatment, comprising 4 biological replicates with 3 larvae each.

Cellular apoptosis was assessed in 5 dpf larvae exposed to isolated CPF, PFHxS, or their combination using the acridine orange assay (Figure S2). No significant differences were observed among treatment groups compared to the solvent control (F_(10, 109)_ = 1.29, *p* = 0.24), Figure 5.

### 3.5. Gene Expression Analysis

**Figure 6:**
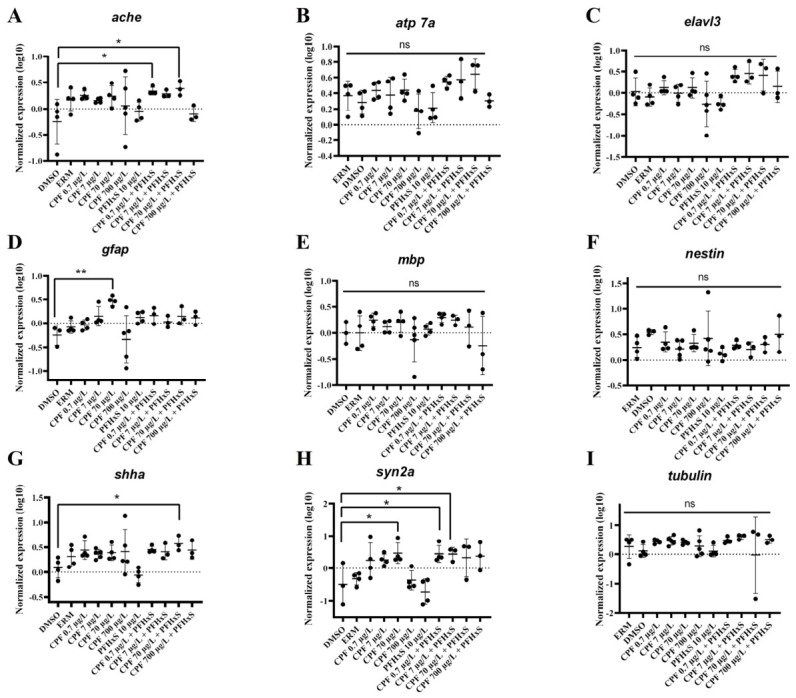
Expression levels of transcripts related to neurotoxicity of zebrafish larvae continuously exposed to chlorpyrifos (CPF) and perfluorohexanesulfonic acid (PFHxS) for five days. (**A**) *ache*, acetylcholinesterase; (**B**) *atp7a*, ATPase copper transporting alpha; (**C**) *elavl3*, ELAV-like neuron-specific RNA binding protein 3; (**D**) *gfap*, glial fibrillary acidic protein; (**E**) *mbp*, myelin basic protein; (**F**) *nestin*, nestin intermediate filament protein; (**G**) *shha*, sonic hedgehog a; (**H**) *syn2a*, synapsin IIa; (**I**) α1-*tubulin*, alpha tubulin (neuronal cytoskeletal protein). Data were analyzed by one-way ANOVA followed by Dunnett’s post hoc test for comparisons with the solvent control. ns = not significant, * *p* ≤ 0.05, ** *p* ≤ 0.01, *n* = 3–5 biological replicates/treatment.

We assessed the expression of genes related to neurotoxicity following exposure to CPF and PFHxS (Figure 6). Transcript abundance did not vary compared with the solvent control for the genes *atp7a* (F_(10,30)_ = 2.614, *p* = 0.0204), *elavl3* (F_(10,31)_ = 2.678, *p* = 0.0173), *mbp* (F_(10,30)_ = 1.322, *p* = 0.2640), *nestin* (F_(10,31)_ = 0.9129, *p* = 0.5338), and *tubulin* (F_(10,32)_ = 0.8885, *p* = 0.5537). Increased abundance of *ache*, compared to the solvent control, was detected following exposure to CPF at 0.7 and 70 µg/L combined with PFHxS (F_(10,31)_ = 1.975, *p* = 0.0719). Similarly, transcript levels of *gfap* were higher in the CPF at 70 µg/L group (F_(10,29)_ = 3.611, *p* = 0.0033). CPF at 70 µg/L combined with PFHxS induced upregulation of *shha* (F_(10,32)_ = 2.572, *p* = 0.0207). A similar trend was observed for *syn2a*, with increased levels in CPF at 70 µg/L as well as when combined with PFHxS (F_(10,29)_ = 4.802, *p* = 0.0004).

**Figure 7:**
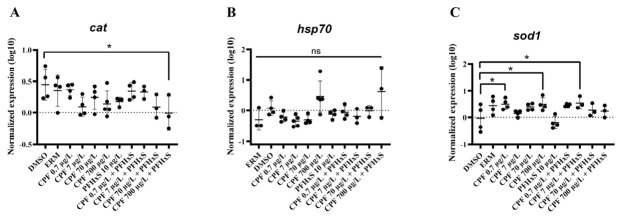
Expression levels of transcripts related to oxidative stress of zebrafish larvae continuously exposed to chlorpyrifos (CPF) and perfluorohexanoic acid (PFHxA) for five days. (**A**) *cat*, catalase; (**B**) *hsp70*, heat shock protein 70; (**C**) *sod1*, superoxide dismutase 1. Data were analyzed by one-way ANOVA followed by Dunnett’s post hoc test for comparisons with the solvent control. ns = not significant, * *p* ≤ 0.05, *n* = 3–5 biological replicates/treatment.

Expression of genes related to oxidative stress was also assessed in zebrafish larvae exposed to CPF and PFHxS (Figure 7). There were no differences in *hsp70* compared to the solvent control (F_(10,31)_ = 3.453, *p* = 0.0038). Exposure to CPF at 700 µg/L combined with PFHxS reduced the transcript abundance of *cat* (F_(10,31)_ = 2.224, *p* = 0.0433). Transcript levels of *sod1* were upregulated in CPF at 0.7 and 700 µg/L, as well as when combined with PFHxS (F_(10,30)_ = 3.508, *p* = 0.0037).

## 4. Discussion

Chlorpyrifos is an organophosphorus pesticide known to cause adverse effects in various non-target aquatic organisms [18,45]. Joint toxicity studies have been conducted with CPF and other pesticides, heavy metals, and microplastics. The outcomes revealed a range of effects, from antagonistic to synergistic toxicity [46,47,48,49]. With PFAS, on the other hand, the combined effects remain poorly investigated, requiring further studies to elucidate their interaction mechanisms and potential ecological risks [31]. In our study, PFHxS modulated CPF toxicity, mostly increasing its effects on mortality, behavior, and expression of genes related to neurotoxicity and oxidative stress.

Exposure to CPF is linked to developmental impairments in human, rodent, avian, and fish models. Particularly in fish models, CPF exposure induced mortality, deformities such as pericardial and yolk sac edema, cardiac apoptosis, and skeletal deformities, as well as alterations in hatchability [50]. In our study, increased mortality was observed in the groups exposed to CPF at 0.7 μg/L (isolated) and 700 μg/L, both alone and in combination with PFHxS. In the combination group, survival decreased drastically to about 5% at 7 dpf. Regarding deformities, isolated CPF increased malformations in the 70 and 700 µg/L groups, whereas in the combination group, this effect was observed only at the highest concentration. Previous studies have already shown the detrimental effects of these chemicals on survival and deformities [16,51,52]. For instance, zebrafish embryos exposed to 10 μM PFHxS from 0 to 1 hpf exhibited a mortality rate considerably higher (20%) than that of the control group (5%). In our study, no differences were found between the control and isolated PFHxS at a much lower concentration (~0.025 µM). For CPF, the LC_50_ estimated for zebrafish larvae is 0.41 mg/L (=410 µg/L) [53], which agrees with our data that showed a 60% reduction in survival at 700 µg/L compared to the control. These authors have also shown that binary mixtures of CPF and the pesticide lambda-cyhalothrin displayed synergic mortality results [49]. Together, these findings reinforce the importance of assessing mixture toxicity, as combined exposures may lead to more pronounced effects on survival than single-compound exposures.

Joint toxicity involving CPF is shaped by multiple factors, such as the mode of action of co-occurring compounds, their interaction potential, and toxicokinetic and toxicodynamic processes and bioaccumulation. As a result, mixture effects can vary widely, ranging from synergistic to antagonistic, depending on the specific context [29,31,54]. A study conducted with CPF and perfluorohexanoic acid (PFHxA) showed, in silico, a possible physicochemical interaction between these two compounds due to opposing partial charges in their structures, which can result in reversible interactions in solution and reduced bioavailability [31,55]. This may partly explain the modest effects on increased mortality and why deformities were not exacerbated under co-exposure. Another factor is the distinct modes of action of these two compounds. CPF acts primarily through the inhibition of acetylcholinesterase following P_450_-dependent bioactivation of CPF to CPF-oxon [44]. PFHxS, on the other hand, may interact with estrogen and androgen receptors, induce oxidative stress, and disrupt lipid metabolism [5]. Thus, physicochemical interactions, together with differences in modes of action, may, at least in part, explain the lack of synergistic effects observed in our study.

In addition to survival, behavioral endpoints provide a sensitive means to detect sublethal effects, as even subtle impairments can have important consequences for organismal fitness [56]. In our study, exposure to CPF and PFHxS, both alone and in combination, induced behavioral changes in zebrafish larvae, mostly evidenced by hypoactivity. Hyperactivity was only observed in larvae exposed to 7 μg/L CPF in the third dark cycle. Konig et al. [57] states that increased locomotor activity may be a result of the irritant effects of chemicals, particularly at low concentrations, which trigger an innate immune response in the animals. Altered expression of genes related to neurotoxicity, mainly motor activity genes (e.g., “motor activity”, “actin binding”, and “ATPase activity”), is also likely to affect behavioral responses in the larvae [58]. Regarding hypoactivity, zebrafish exposed to 100 ng/mL CPF also showed similar responses at 6 and 9 hpf [59]. In rat models, 1 mg/kg/mL of CPF administered by oral gavage from postnatal day 10 to 15 resulted in decreased motricity in both male and female Wistar rats during late adulthood. These authors postulated that CPF can induce a biphasic response in the animals, characterized by an initial increase in motor activity followed by a progressive decline in motility [60].

Both responses of hypoactivity and hyperactivity were also detected in zebrafish larvae exposed continuously to CPF and PFHxA for seven days [31]. These authors suggest that increased AChE activity in the combination group may contribute to altered locomotor behavior, possibly reflecting the transition from initial hyperactivity to reduced activity. In the present study, increased transcript abundance of *ache* was only detected in the combination group with CPF at 0.7 and 70 μg/L and PFHxS at 10 μg/L, which suggests that PFHxS may increase CPF neurotoxicity in zebrafish larvae. Synergistic inhibition of AChE activity was detected in the brain of carp (*Cyprinus carpio*) exposed to CPF and malathion, which is also an organophosphorus pesticide [61]. Similar results were also observed in developing zebrafish exposed to mixtures of CPF and the s-triazine herbicides atrazine and terbuthylazine. Such synergistic interactions may involve enhanced conversion of CPF into its more toxic oxon metabolite, possibly due to co-exposure-induced activation of cytochrome P_450_ enzymes [49].

CPF exposure has also been linked to neurotoxicity and behavioral impairments through mechanisms independent of AChE inhibition [62]. Indeed, in our study, transcript levels of *gfap*, *shha*, and *syn2a* were upregulated following exposure to CPF alone (*gfap* and *syn2a*) and to CPF combined with PFHxS (*shha* and *syn2a*). GFAP (glial fibrillary acidic protein) is an intermediate filament protein predominantly expressed in astrocytes, where it contributes to cytoskeletal organization and stability [63]. Increased transcript levels of *gfap* were also observed in zebrafish exposed to GenX, a chemical used to replace the perfluorooctanoic acid (PFOA), as well as CPF combined with PFHxA [26,64]. These authors argue that *gfap* expression is often assessed as a marker of neurotoxicity, and its upregulation may indicate an adaptive response aimed at maintaining neuronal–glial interactions and structural integrity in response to exposure. Sonic hedgehog a (*shha*) is a critical signaling molecule governing neural development and progenitor cell activity [65]. Its upregulation in the combined exposure group may reflect a compensatory response, suggesting activation of developmental and regenerative pathways. Similar results were also found in zebrafish larvae exposed to 300 μg/L CPF, suggesting that CPF may disrupt neurodevelopmental signaling pathways, which may in turn contribute to altered behavior [52]. Finally, *syn2a* encodes a synaptic vesicle-associated protein involved in maintaining the reserve pool of vesicles and regulating neurotransmitter release [66]. Its upregulation was also observed in developing zebrafish exposed to rotenone (5 μg/L) and may reflect alterations in synaptic activity and neurotransmission [67]. Collectively, these data highlight the neurotoxic effects of CPF, which may be enhanced by co-exposure with PFHxS. Future transcriptomic analyses could provide further insight into the mechanisms underlying these effects.

In addition to neurotoxicity, oxidative stress plays a central role in mediating the toxic effects of pesticides in aquatic organisms [68,69,70]. In our study, the transcript abundance of *cat* was downregulated in the co-exposure group, whereas increased levels of *sod1* transcripts were found following exposure to CPF alone and combined with PFHxS. However, there were no differences in ROS levels in any of the treatment groups. Superoxide dismutase (SOD), which constitutes the first line of antioxidant defense, catalyzes the dismutation of superoxide radicals into hydrogen peroxide, which is subsequently detoxified by catalase (CAT) [71]. We hypothesize that the observed transcriptional changes may reflect an early or compensatory antioxidant response that is sufficient to maintain basal ROS homeostasis under the tested conditions. Different studies, on the other hand, have reported that exposure to CPF, either alone or in combination with other contaminants, can induce antioxidant defense responses in zebrafish [31,72,73]. However, it is important to note that the expression of genes related to oxidative stress may vary depending on several factors, including developmental stage, exposure duration, concentration, tissue specificity, and the presence of co-contaminants [74,75,76]. Additionally, the lack of changes in ROS levels observed in the present study may reflect the dynamic and tightly regulated nature of redox homeostasis. This is further supported by the absence of significant differences in apoptosis observed both in the present study and in previous reports, suggesting that the antioxidant response may have been effective in preventing downstream cellular damage under these conditions [31]. However, we cannot exclude the possibility that subtle apoptotic responses were not detected by acridine orange staining, and future studies employing more sensitive methods, such as TUNEL assays or transmission electron microscopy, would help to further evaluate this possibility. Finally, it is also important to highlight that we did not assess the activity of antioxidant enzymes or oxidative damage markers, such as lipid peroxidation and protein carbonyl content. This represents a limitation of the present study and should be addressed in future investigations to provide a more comprehensive understanding of oxidative stress responses.

## 5. Conclusions

In conclusion, CPF, both alone and in combination with PFHxS, induced mortality and developmental deformities in zebrafish. However, the contribution of PFHxS to CPF toxicity, based on these endpoints, appeared to be limited. Behavioral alterations were also observed, with reduced locomotor activity detected at concentrations as low as CPF 0.7 µg/L combined with PFHxS 10 µg/L. These effects are consistent with the known neurotoxic potential of CPF and were supported by changes in the expression of genes associated with neurotoxicity (e.g., *ache*, *shha*, *syn2a*) in both single and combined exposures. Effects on the expression of these genes were also detected at concentrations as low as CPF 0.7 µg/L combined with PFHxS 10 µg/L. In addition, genes related to oxidative stress were differentially expressed, although no changes in ROS levels were detected, suggesting that the antioxidant response may have been sufficient to maintain redox homeostasis under the tested conditions. Overall, these findings indicate that CPF is a major driver of the observed toxic effects, while PFHxS exerts a comparatively minor modulatory role. Few studies explore the mixing effects of PFASs with other pollutants in the environment [51]. Future studies should investigate the long-term effects of CPF and PFHxS co-exposure on zebrafish development, oxidative stress, and behavior, particularly under chronic exposure to environmentally relevant concentrations, as well as explore additional molecular and biochemical endpoints to better understand the underlying mechanisms of toxicity.

## Data Availability

The raw data supporting the conclusions of this article will be made available by the authors on request.

## Supplementary Materials

The following supporting information can be downloaded at: https://www.mdpi.com/article/10.3390/toxics14070566/s1.

## References

[1] Samarasinghe V.A.C., Bahar M.M., Qi F., Yan K., Liu Y., Naidu R. (2023). Evaluating PFHxS toxicity to invertebrates and microbial processes in soil. Environ. Chem. Ecotoxicol..

[2] Wang Z., Buser A.M., Cousins I.T., Demattio S., Drost W., Johansson O., Ohno K., Patlewicz G.Y., Richard A.M., Walker G.W. (2021). A new OECD definition for per- and polyfluoroalkyl substances. Environ. Sci. Technol..

[3] Buck R.C., Murphy P.M., Pabon M., Knepper T.P., Lange F.T. (2012). Chemistry properties use of commercial fluorinated surfactants. The Handbook of Environmental Chemistry: Polyfluorinated Chemicals and Transformation Products.

[4] Ng C., Cousins I.T., DeWitt J.C., Glüge J., Goldenman G., Herzke D., Lohmann R., Miller M., Patton S., Scheringer M. (2021). Addressing urgent questions for PFAS in the 21st century. Environ. Sci. Technol..

[5] Kreychman M., Ivantsova E., Lu A., Bisesi J.H., Martyniuk C.J. (2024). A comparative review of the toxicity mechanisms of perfluorohexanoic acid (PFHxA) and perfluorohexanesulphonic acid (PFHxS) in fish. Comp. Biochem. Physiol. Part C Toxicol. Pharmacol..

[6] Sultan A., Konig I., Point A.D., Buttrós V.H., Lewis R.L., Simmons D.B.D., Martyniuk C.J. (2025). Dietary exposure to hexafluoropropylene oxide dimer acid (HFPO-DA ammonium salt, GenX) alters hepatic sphingomyelin and phosphatidylcholine metabolism in Nile Tilapia (*Oreochromis niloticus*). Aquat. Toxicol..

[7] Cai H., Lin Q., Gong C., Yu F., Jin L., Peng R. (2026). PFAS-induced immunotoxicity in freshwater fish of inland China: Mechanisms and ecological risks. Comp. Biochem. Physiol. Part C Toxicol. Pharmacol..

[8] Zhong H., Liu W., Li N., Ma D., Zhao C., Li J., Wang Y., Jiang G. (2022). Assessment of perfluorohexane sulfonic acid (PFHxS)-related compounds degradation potential: Computational and experimental approaches. J. Hazard. Mater..

[9] Wang Z., Cousins I.T., Scheringer M., Hungerbühler K. (2013). Fluorinated alternatives to long-chain perfluoroalkyl carboxylic acids (PFCAs), perfluoroalkane sulfonic acids (PFSAs) and their potential precursors. Environ. Int..

[10] Babayev M., Capozzi S.L., Miller P., McLaughlin K.R., Seguinot Medina S., Byrne S., Zheng G., Salamova A. (2022). PFAS in drinking water and serum of the people of a southeast Alaska community: A pilot study. Environ. Pollut..

[11] Nguyen V.T., Reinhard M., Karina G.Y.-H. (2011). Occurrence and source characterization of perfluorochemicals in an urban watershed. Chemosphere.

[12] Cui Q., Pan Y., Zhang H., Sheng N., Dai J. (2018). Elevated concentrations of perfluorohexanesulfonate and other per- and polyfluoroalkyl substances in Baiyangdian Lake (China): Source characterization and exposure assessment. Environ. Pollut..

[13] Anderson R.H., Long G.C., Porter R.C., Anderson J.K. (2016). Occurrence of select perfluoroalkyl substances at, U.S. Air Force aqueous film-forming foam release sites other than fire-training areas: Field-validation of critical fate and transport properties. Chemosphere.

[14] Sands M., Zhang X., Jensen T., La Frano M., Lin M., Irudayaraj J. (2024). PFAS assessment in fish—Samples from Illinois waters. Sci. Total Environ..

[15] Runkel A.A., Stajnko A., Snoj Tratnik J., Mazej D., Horvat M., Přibylová P., Kosjek T. (2023). Exposure of children and adolescents from Northeastern Slovenia to per- and polyfluoroalkyl substances. Chemosphere.

[16] Ulhaq Z.S., Tse W.K.F. (2023). Perfluorohexanesulfonic acid (PFHxS) induces oxidative stress and causes developmental toxicities in zebrafish embryos. J. Hazard. Mater..

[17] Ansari I., El-Kady M.M., Arora C., Sundararajan M., Maiti D., Khan A. (2021). A review on the fatal impact of pesticide toxicity on environment and human health. Global Climate Change.

[18] Wołejko E., Łozowicka B., Jabłońska-Trypuć A., Pietruszyńska M., Wydro U. (2022). Chlorpyrifos occurrence and toxicological risk assessment: A review. Int. J. Environ. Res. Public Health.

[19] Echeverri-Jaramillo G., Jaramillo-Colorado B., Sabater-Marco C., Castillo-López M.-Á. (2021). Cytotoxic and estrogenic activity of chlorpyrifos and its metabolite 3,5,6-trichloro-2-pyridinol: Study of marine yeasts as potential toxicity indicators. Ecotoxicology.

[20] Arain M., Brohi K.M., Channa A., Brohi R.O.Z., Mushtaque S., Kumar K., Sameeu A. (2018). Analysis of chlorpyrifos pesticide residues in surface water, ground water and vegetables through gas chromatography. J. Int. Environ. Appl. Sci..

[21] Rico A., de Oliveira R., Silva de Souza Nunes G., Rizzi C., Villa S., De Caroli Vizioli B., Montagner C.C., Waichman A.V. (2022). Ecological risk assessment of pesticides in urban streams of the Brazilian Amazon. Chemosphere.

[22] Wan Y., Tran T.M., Nguyen V.T., Wang A., Wang J., Kannan K. (2021). Neonicotinoids, fipronil, chlorpyrifos, carbendazim, chlorotriazines, chlorophenoxy herbicides, bentazon, and selected pesticide transformation products in surface water and drinking water from northern Vietnam. Sci. Total Environ..

[23] Huang X., Cui H., Duan W. (2020). Ecotoxicity of chlorpyrifos to aquatic organisms: A review. Ecotoxicol. Environ. Saf..

[24] Gómez-Canela C., Prats E., Piña B., Tauler R. (2017). Assessment of chlorpyrifos toxic effects in zebrafish (*Danio rerio*) metabolism. Environ. Pollut..

[25] Ma J., Zhu P., Wang W., Zhang X., Wang P., Sultan Y., Li Y., Ding W., Li X. (2023). Environmental impacts of chlorpyrifos: Transgenerational toxic effects on aquatic organisms cannot be ignored. Sci. Total Environ..

[26] Richendrfer H., Pelkowski S.D., Colwill R.M., Créton R. (2012). Developmental sub-chronic exposure to chlorpyrifos reduces anxiety-related behavior in zebrafish larvae. Neurotoxicol. Teratol..

[27] Jiang J., He B., Wei Y., Cui J., Zhang Q., Liu X., Liu D., Wang P., Zhou Z. (2022). The toxic effects of combined exposure of chlorpyrifos and p,p’-DDE to zebrafish (*Danio rerio*) and tissue bioaccumulation. Aquat. Toxicol..

[28] Zhang W., Fan R., Luo S., Liu Y., Jin Y., Li Y., Xiong M., Yuan X., Jia L., Chen Y. (2022). Antagonistic effects and mechanisms of carbendazim and chlorpyrifos on the neurobehavior of larval zebrafish. Chemosphere.

[29] Zhang W., Fan R., Luo S., Liu Y., Jin Y., Li Y., Li B., Chen Y., Jia L., Yuan X. (2022). Combined effects of chlorpyrifos and cyfluthrin on neurobehavior and neurotransmitter levels in larval zebrafish. J. Appl. Toxicol..

[30] Kienle C., Köhler H.-R., Gerhardt A. (2009). Behavioural and developmental toxicity of chlorpyrifos and nickel chloride to zebrafish (*Danio rerio*) embryos and larvae. Ecotoxicol. Environ. Saf..

[31] Valle E.M.A., Sultan A., Konig I., Cabello C.Q., Oliveira H.P.M., Codognoto L., Martyniuk C.J. (2025). Toxicity assessment of mixture effects of insecticides and perfluorinated chemicals (PFAS) in zebrafish (*Danio rerio*): A case study with chlorpyrifos and perfluorohexanoic acid (PFHxA). J. Appl. Toxicol..

[32] Westerfield M. (2000). The Zebrafish Book: A Guide for the Laboratory Use of Zebrafish. http://zfin.org/zf_info/zfbook/zfbk.html.

[33] Kimmel C.B., Ballard W.W., Kimmel S.R., Ullmann B., Schilling T.F. (1995). Stages of embryonic development of the zebrafish. Dev. Dyn..

[34] Qiao K., Hu T., Jiang Y., Huang J., Hu J., Gui W., Ye Q., Li S., Zhu G. (2021). Crosstalk of cholinergic pathway on thyroid disrupting effects of the insecticide chlorpyrifos in zebrafish (*Danio rerio*). Sci. Total Environ..

[35] Ivantsova E., Konig I., Lopez-Scarim V., English C., Charnas S.R., Souders C.L., Martyniuk C.J. (2023). Molecular and behavioral toxicity assessment of tiafenacil, a novel PPO-inhibiting herbicide in zebrafish embryos/larvae. Environ. Toxicol. Pharmacol..

[36] David N., Ivantsova E., Konig I., English C.D., Avidan L., Kreychman M., Rivera M.L., Escobar C., Valle E.M.A., Sultan A. (2024). Adverse Outcomes Following Exposure to Perfluorooctanesulfonamide (PFOSA) in Larval Zebrafish (*Danio rerio*): A Neurotoxic and Behavioral Perspective. Toxics.

[37] Patel N., Ivantsova E., Konig I., Souders C.L., Martyniuk C.J. (2022). Perfluorotetradecanoic Acid (PFTeDA) Induces Mitochondrial Damage and Oxidative Stress in Zebrafish (*Danio rerio*) Embryos/Larvae. Toxics.

[38] Wang X.H., Souders C.L., Zhao Y.H., Martyniuk C.J. (2018). Paraquat affects mitochondrial bioenergetics, dopamine system expression, and locomotor activity in zebrafish (*Danio rerio*). Chemosphere.

[39] Zucchi S., Blüthgen N., Ieronimo A., Fent K. (2011). The UV-absorber benzophenone-4 alters transcripts of genes involved in hormonal pathways in zebrafish (*Danio rerio*) eleuthero-embryos and adult males. Toxicol. Appl. Pharmacol..

[40] Sarkar S., Mukherjee S., Chattopadhyay A., Bhattacharya S. (2014). Low dose of arsenic trioxide triggers oxidative stress in zebrafish brain: Expression of antioxidant genes. Ecotoxicol. Environ. Saf..

[41] Lin C.T., Tseng W.C., Hsiao N.W., Chang H.H., Ken C.F. (2009). Characterization, molecular modelling and developmental expression of zebrafish manganese superoxide dismutase. Fish Shellfish Immunol..

[42] Yang Q., Deng P., Xing D., Liu H., Shi F., Hu L., Zou X., Nie H., Zuo J., Zhuang Z. (2023). Developmental Neurotoxicity of Difenoconazole in Zebrafish Embryos. Toxics.

[43] Jiang F., Liu J., Zeng X., Yu L., Liu C., Wang J. (2018). Tris (2-butoxyethyl) phosphate affects motor behavior and axonal growth in zebrafish (*Danio rerio*) larvae. Aquat. Toxicol..

[44] Guo Y., Fu Y., Sun W. (2023). 50 Hz Magnetic Field Exposure Inhibited Spontaneous Movement of Zebrafish Larvae through ROS-Mediated syn2a Expression. Int. J. Mol. Sci..

[45] Bhende R.S., Jhariya U., Srivastava S., Bombaywala S., Das S.B., Dafale N.A. (2022). Environmental distribution, metabolic fate, and degradation mechanism of chlorpyrifos: Recent and future perspectives. Appl. Biochem. Biotechnol..

[46] Sun M., Zhao Y., Qi S., Ye C., Zhang J., Fei C., Li J., Zhou S., Wu D. (2024). Evaluation of the combined toxicity of heavy metals and chlorpyrifos: A comparison of electrochemical and MTT methods. Electroanalysis.

[47] Huang Z., Xiao X., Wang D., Zhong Y., Ding Q., You J. (2023). Joint effects of micro-sized polystyrene and chlorpyrifos on zebrafish based on multiple endpoints and gut microbial effects. J. Environ. Sci..

[48] Kunwar P.S., Sapkota B., Badu S., Kandel S., Pandey R. (2022). Chlorpyrifos and dichlorvos in combined exposure reveals antagonistic interaction to the freshwater fish *Cirrhinus mrigala*. Ecotoxicology.

[49] Pérez J., Domingues I., Monteiro M., Soares A.M.V.M., Loureiro S. (2013). Synergistic effects caused by atrazine and terbuthylazine on chlorpyrifos toxicity to early-life stages of the zebrafish *Danio rerio*. Environ. Sci. Pollut. Res..

[50] Coppola L., Lori G., Tait S., Sogorb M.A., Estevan C. (2025). Evaluation of developmental toxicity of chlorpyrifos through new approach methodologies: A systematic review. Arch. Toxicol..

[51] Jin Y., Liu Z., Peng T., Fu Z. (2015). The toxicity of chlorpyrifos on the early life stage of zebrafish: A survey on the endpoints at development, locomotor behavior, oxidative stress and immunotoxicity. Fish Shellfish Immunol..

[52] Yu K., Li G., Feng W., Liu L., Zhang J., Wu W., Xu L., Yan Y. (2015). Chlorpyrifos is estrogenic and alters embryonic hatching, cell proliferation and apoptosis in zebrafish. Chem.-Biol. Interact..

[53] Shen W., Lou B., Xu C., Yang G., Yu R., Wang X., Li X., Wang Q., Wang Y. (2020). Lethal toxicity and gene expression changes in embryonic zebrafish upon exposure to individual and mixture of malathion, chlorpyrifos and lambda-cyhalothrin. Chemosphere.

[54] Cedergreen N. (2014). Quantifying synergy: A systematic review of mixture toxicity studies within environmental toxicology. PLoS ONE.

[55] Valle E.M.A., Ivantsova E., Pracchia M.L., Cabello C.Q., de Oliveira H.P.M., Codognoto L., Martyniuk C.J. (2026). Do Perfluorinated Chemicals Enhance the Toxicity of Other Contaminants in Aquatic Organisms? A Review. Toxics.

[56] Tierney K.B. (2011). Behavioural assessments of neurotoxic effects and neurodegeneration in zebrafish. Biochim. Biophys. Acta (BBA)—Mol. Basis Dis..

[57] Konig I., Iftikhar N., Henry E., English C., Ivantsova E., Souders C.L., Marcussi S., Martyniuk C.J. (2023). Toxicity assessment of carvacrol and its acetylated derivative in early staged zebrafish (*Danio rerio*): Safer alternatives to fipronil-based pesticides?. Comp. Biochem. Physiol. Part C Toxicol. Pharmacol..

[58] Chen X., Zheng J., Teng M., Zhang J., Qian L., Duan M., Cheng Y., Zhao W., Wang Z., Wang C. (2022). Tralopyril affects locomotor activity of zebrafish (*Danio rerio*) by impairing tail muscle tissue, the nervous system, and energy metabolism. Chemosphere.

[59] Levin E.D., Swain H.A., Donerly S., Linney E. (2004). Developmental chlorpyrifos effects on hatchling zebrafish swimming behavior. Neurotoxicol. Teratol..

[60] Perez-Fernandez C., Morales-Navas M., Guardia-Escote L., Colomina M.T., Gimenez E., Sanchez-Santed F. (2021). Pesticides and aging: Preweaning exposure to chlorpyrifos induces a general hypomotricity state in late-adult rats. Neurotoxicology.

[61] Chen C., Wang Y., Zhao X., Wang Q., Qiu L. (2014). The combined toxicity assessment of carp (*Cyprinus carpio*) acetylcholinesterase activity by binary mixtures of chlorpyrifos and four other insecticides. Ecotoxicology.

[62] Eaton D.L., Daroff R.B., Autrup H., Bridges J., Buffler P., Costa L.G., Coyle J., McKhann G., Mobley W.C., Nadel L. (2008). Review of the toxicology of chlorpyrifos with an emphasis on human exposure and neurodevelopment. Crit. Rev. Toxicol..

[63] Nielsen A.L., Jørgensen A.L. (2003). Structural and functional characterization of the zebrafish gene for glial fibrillary acidic protein, GFAP. Gene.

[64] Ivantsova E., Lopez-Scarim V., Sultan A., English C., Biju A., Souders C.L., Padillo-Anthemides N.E., Konig I., Martyniuk C.J. (2023). Evidence for neurotoxicity and oxidative stress in zebrafish embryos/larvae treated with HFPO-DA ammonium salt (GenX). Environ. Toxicol. Pharmacol..

[65] Reimer M.M., Kuscha V., Wyatt C., Sörensen I., Frank R.E., Knüwer M., Becker T., Becker C.G. (2009). Sonic hedgehog is a polarized signal for motor neuron regeneration in adult zebrafish. J. Neurosci..

[66] Longhena F., Faustini G., Brembati V., Pizzi M., Bellucci A. (2021). An updated reappraisal of synapsins: Structure, function and role in neurological and psychiatric disorders. Neurosci. Biobehav. Rev..

[67] Palaniselvam S., Narasimman V., Vijayashree R., Ramachandran S. (2025). Neuroprotective effect of nano-carboxymethyl chitosan from Doryteuthis sibogae against rotenone-induced Parkinson’s disease in the zebrafish model. Behav. Brain Res..

[68] Chowdhury S., Saikia S.K. (2022). Use of zebrafish as a model organism to study oxidative stress: A review. Zebrafish.

[69] Sule R.O., Condon L., Gomes A.V. (2022). A common feature of pesticides: Oxidative stress—The role of oxidative stress in pesticide-induced toxicity. Oxidative Med. Cell. Longev..

[70] Rodríguez-Fuentes G., Rubio-Escalante F.J., Noreña-Barroso E., Escalante-Herrera K.S., Schlenk D. (2015). Impacts of oxidative stress on acetylcholinesterase transcription, and activity in embryos of zebrafish (*Danio rerio*) following chlorpyrifos exposure. Comp. Biochem. Physiol. Part C Toxicol. Pharmacol..

[71] Melo N., de Souza S.P., Konig I., de Jesus Paula D.A., Ferreira I.S., Luz R.K., Solis Murgas L.D. (2024). Sensitivity of different organs and tissues as biomarkers of oxidative stress in juvenile tambaqui (*Colossoma macropomum*) submitted to fasting. Comp. Biochem. Physiol. Part A Mol. Integr. Physiol..

[72] Falfushynska H., Khatib I., Kasianchuk N., Lushchak O., Horyn O., Sokolova I.M. (2022). Toxic effects and mechanisms of common pesticides (*Roundup and chlorpyrifos*) and their mixtures in a zebrafish model (*Danio rerio*). Sci. Total Environ..

[73] Wang X., Shen M., Zhou J., Jin Y. (2019). Chlorpyrifos disturbs hepatic metabolism associated with oxidative stress and gut microbiota dysbiosis in adult zebrafish. Comp. Biochem. Physiol. Part C Toxicol. Pharmacol..

[74] van de Pol I.L.E., Hermaniuk A., Verberk W.C.E.P. (2021). Interacting effects of cell size and temperature on gene expression, growth, development and swimming performance in larval zebrafish. Front. Physiol..

[75] Khazaee M., Guardian M.G.E., Aga D.S., Ng C.A. (2020). Impacts of sex and exposure duration on gene expression in zebrafish following perfluorooctane sulfonate exposure. Environ. Toxicol. Chem..

[76] Ito K., Takizawa F., Yoshiura Y., Ototake M., Nakanishi T., Fischer U. (2008). Expression profile of cytokine and transcription factor genes during embryonic development of zebrafish *Danio rerio*. Fish. Sci..

