# Supplementary material for: Neurobehavioral and Molecular Alterations Following Single and Combined Exposure to Chlorpyrifos and PFHxS in Developing Zebrafish (Danio rerio)
Source: Toxics. 2026 Jun 27;14(7):566. doi: 10.3390/toxics14070566 (PMC13418585; doi:10.3390/toxics14070566)
Supplement: Supplementary file 1 [file toxics-14-00566-s001.zip › toxics-4348990 Supplemental File S1 data_qPCR_R1.pdf]

Neurobehavioral and molecular alterations following single and combined exposure to chlorpyrifos and PFHxS in developing zebrafish (*Danio rerio*)

Eliana Maira Agostini Valle [1,2], Amany Sultan [1,3], Michelle Puerta [1], Romanna Shams [1,4], Jack Reites [1], Isaac Konig [1,5], and Christopher J. Martyniuk[1, 6]*

[1] Center for Environmental and Human Toxicology, Department of Physiological Sciences, College of Veterinary Medicine, University of Florida, Gainesville, Florida, 32611, USA

[2] Universidade Federal de São Paulo – Instituto de Ciências Ambientais, Químicas e Farmacêuticas – Campus Diadema – Brasil

[3] Animal Health Research Institute, Agriculture Research Center (ARC), Egypt

[4] University of Veterinary and Animal Sciences, Lahore, Pakistan

[5] Department of Biochemistry, Federal University of Rio Grande do Sul (UFRGS), Porto Alegre, Rio Grande do Sul, Brazil

[6] UF Genetics Institute, Interdisciplinary Program in Biomedical Sciences and Neuroscience, University of Florida, USA

*Correspondence:

Chris Martyniuk, email: cmartyn@ufl.edu

ORCID: 0000-0003-0921-4796

Supplemental results

qPCR- Melt curves and amplification curves

Housekeeping genes (used to normalize the data)

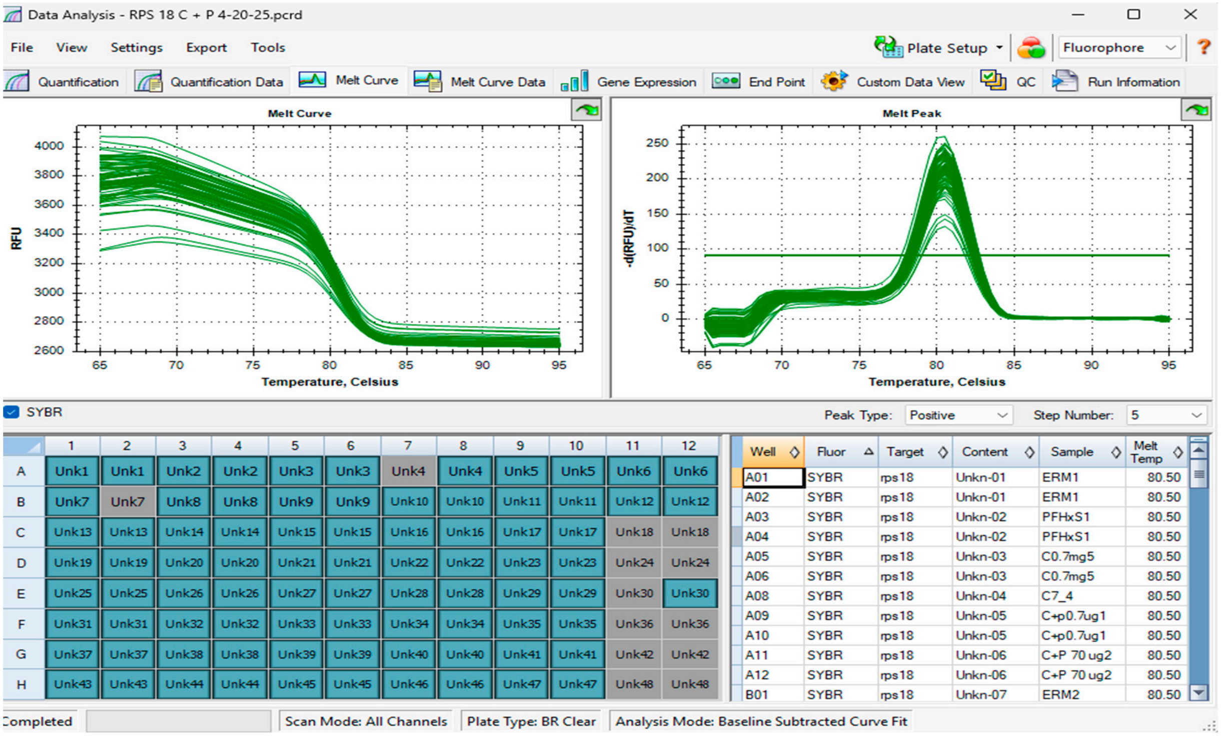

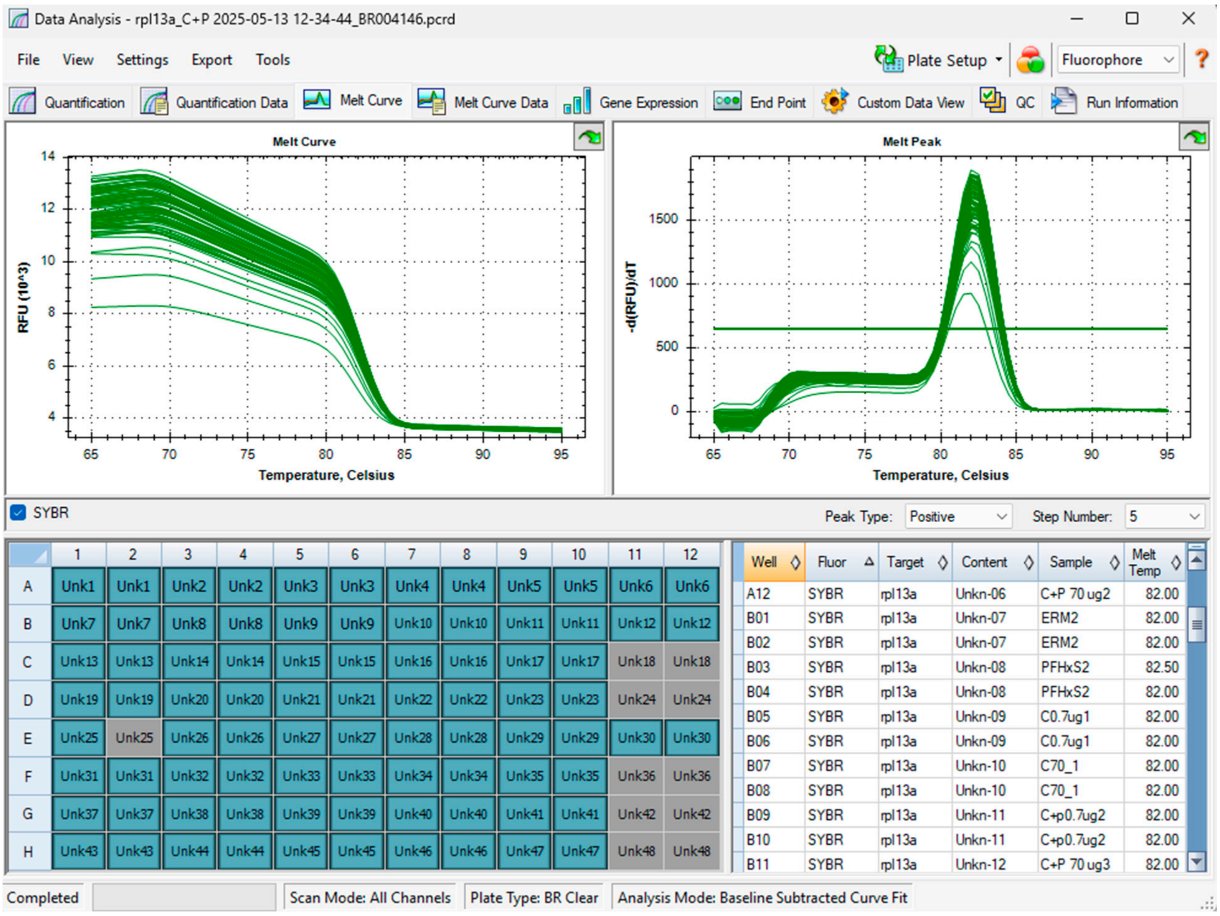
Data Analysis - rpl13a_C+P 2025-05-13 12-34-44_BR004146.pcrd
File View Settings Export Tools
Plate Setup
Fluorophore
Quantification
Quantification Data
Melt Curve
Melt Curve Data
Gene Expression
End Point
Custom Data View
QC
Run Information
Melt Curve
RFU (10^3)
Temperature, Celsius
Melt Peak
-d(RFU)/dT
Temperature, Celsius
SYBR
Peak Type: Positive
Step Number: 5
Well Fluor Target Content Sample Melt Temp
A12 SYBR rpl13a Unkn-06 C+P 70 ug2 82.00
B01 SYBR rpl13a Unkn-07 ERM2 82.00
B02 SYBR rpl13a Unkn-07 ERM2 82.00
B03 SYBR rpl13a Unkn-08 PFHxS2 82.50
B04 SYBR rpl13a Unkn-08 PFHxS2 82.00
B05 SYBR rpl13a Unkn-09 C0.7ug1 82.00
B06 SYBR rpl13a Unkn-09 C0.7ug1 82.00
B07 SYBR rpl13a Unkn-10 C70_1 82.00
B08 SYBR rpl13a Unkn-10 C70_1 82.00
B09 SYBR rpl13a Unkn-11 C+p0.7ug2 82.00
B10 SYBR rpl13a Unkn-11 C+p0.7ug2 82.00
B11 SYBR rpl13a Unkn-12 C+P 70 ug3 82.00
Completed
Scan Mode: All Channels
Plate Type: BR Clear
Analysis Mode: Baseline Subtracted Curve Fit

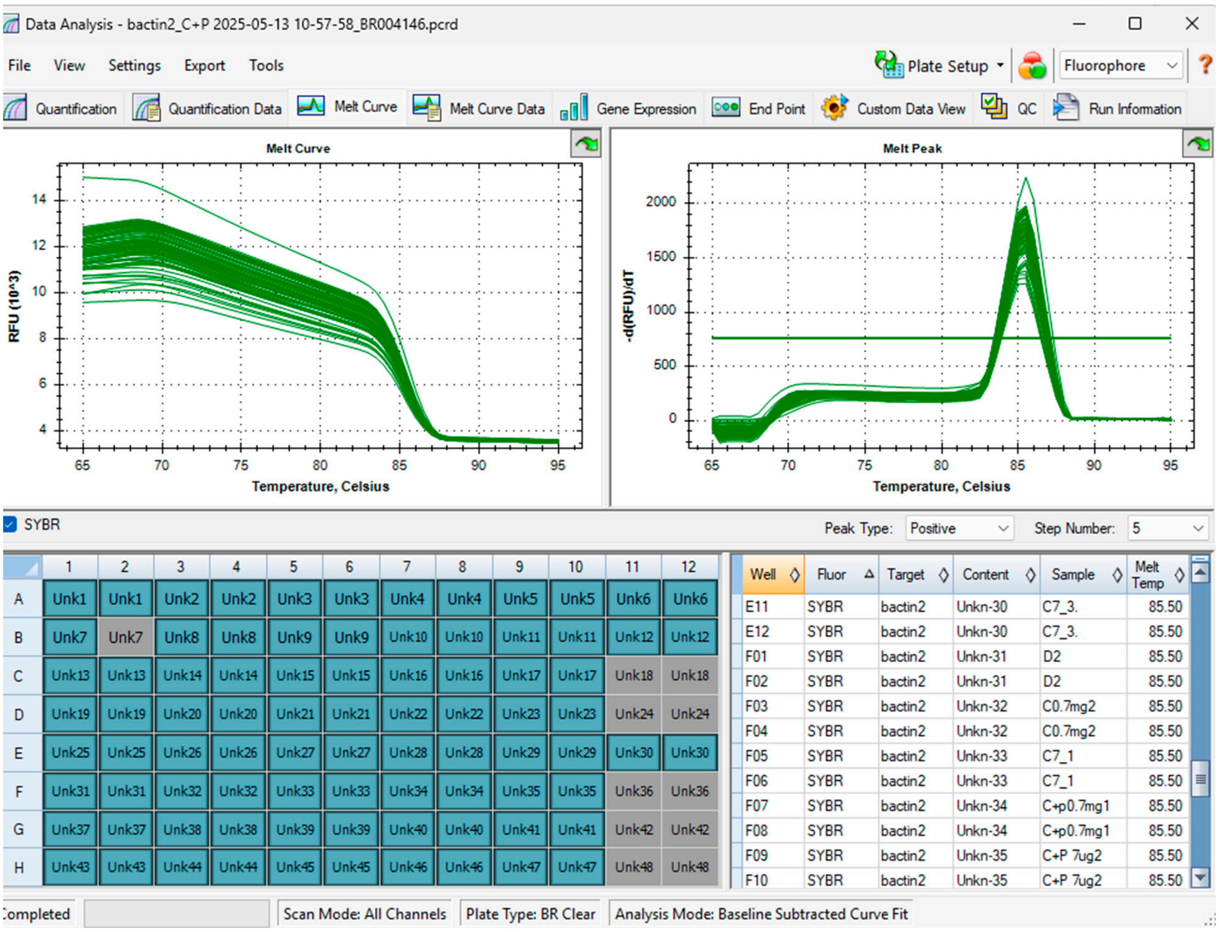

	1	2	3	4	5	6	7	8	9	10	11	12
A	Unk1	Unk1	Unk2	Unk2	Unk3	Unk3	Unk4	Unk4	Unk5	Unk5	Unk6	Unk6
B	Unk7	Unk7	Unk8	Unk8	Unk9	Unk9	Unk10	Unk10	Unk11	Unk11	Unk12	Unk12
C	Unk13	Unk13	Unk14	Unk14	Unk15	Unk15	Unk16	Unk16	Unk17	Unk17	Unk18	Unk18
D	Unk19	Unk19	Unk20	Unk20	Unk21	Unk21	Unk22	Unk22	Unk23	Unk23	Unk24	Unk24
E	Unk25	Unk25	Unk26	Unk26	Unk27	Unk27	Unk28	Unk28	Unk29	Unk29	Unk30	Unk30
F	Unk31	Unk31	Unk32	Unk32	Unk33	Unk33	Unk34	Unk34	Unk35	Unk35	Unk36	Unk36
G	Unk37	Unk37	Unk38	Unk38	Unk39	Unk39	Unk40	Unk40	Unk41	Unk41	Unk42	Unk42
H	Unk43	Unk43	Unk44	Unk44	Unk45	Unk45	Unk46	Unk46	Unk47	Unk47	Unk48	Unk48

Well	Fluor	Target	Content	Sample	Melt Temp
E11	SYBR	bactin2	Unkn-30	C7_3.	85.50
E12	SYBR	bactin2	Unkn-30	C7_3.	85.50
F01	SYBR	bactin2	Unkn-31	D2	85.50
F02	SYBR	bactin2	Unkn-31	D2	85.50
F03	SYBR	bactin2	Unkn-32	C0.7mg2	85.50
F04	SYBR	bactin2	Unkn-32	C0.7mg2	85.50
F05	SYBR	bactin2	Unkn-33	C7_1	85.50
F06	SYBR	bactin2	Unkn-33	C7_1	85.50
F07	SYBR	bactin2	Unkn-34	C+p0.7mg1	85.50
F08	SYBR	bactin2	Unkn-34	C+p0.7mg1	85.50
F09	SYBR	bactin2	Unkn-35	C+P 7ug2	85.50
F10	SYBR	bactin2	Unkn-35	C+P 7ug2	85.50

Genes related to oxidative stress

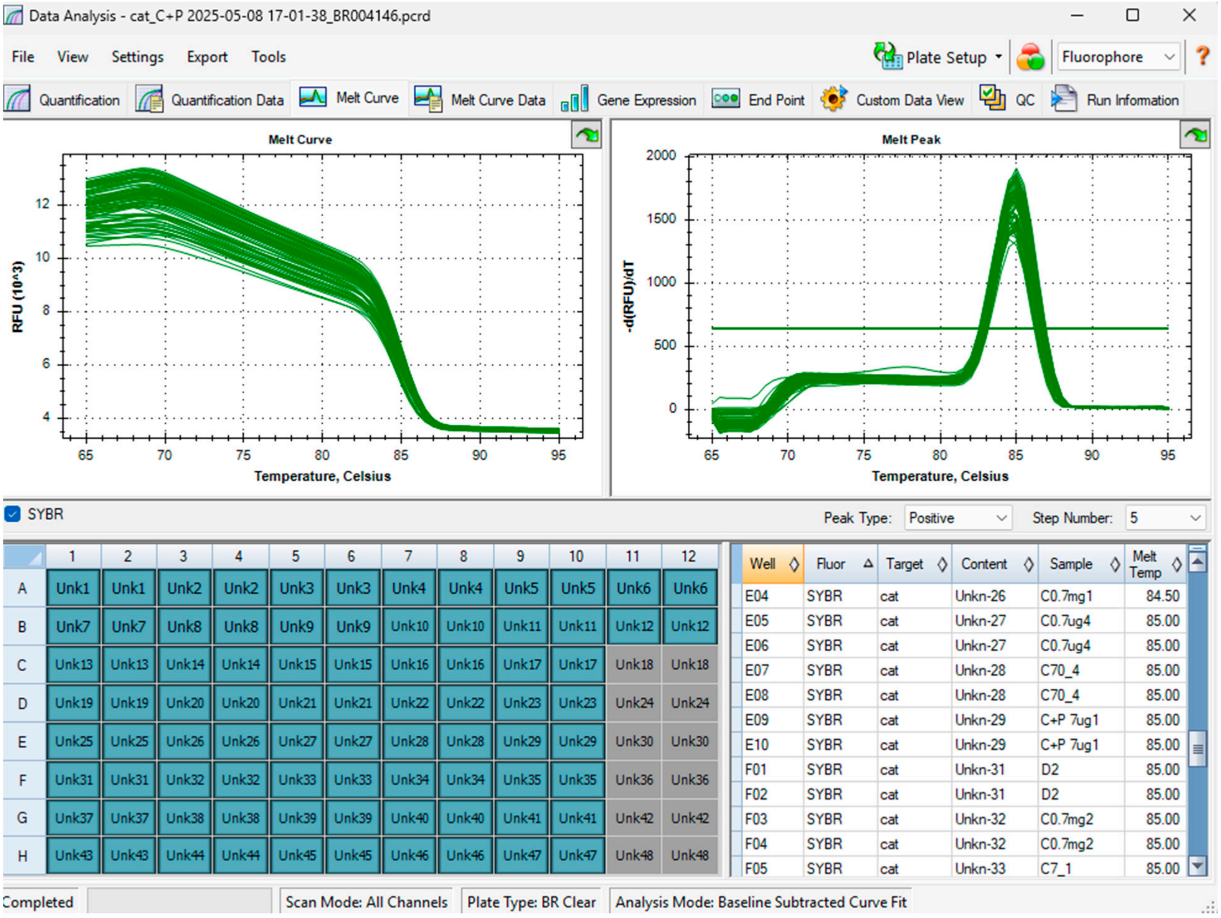

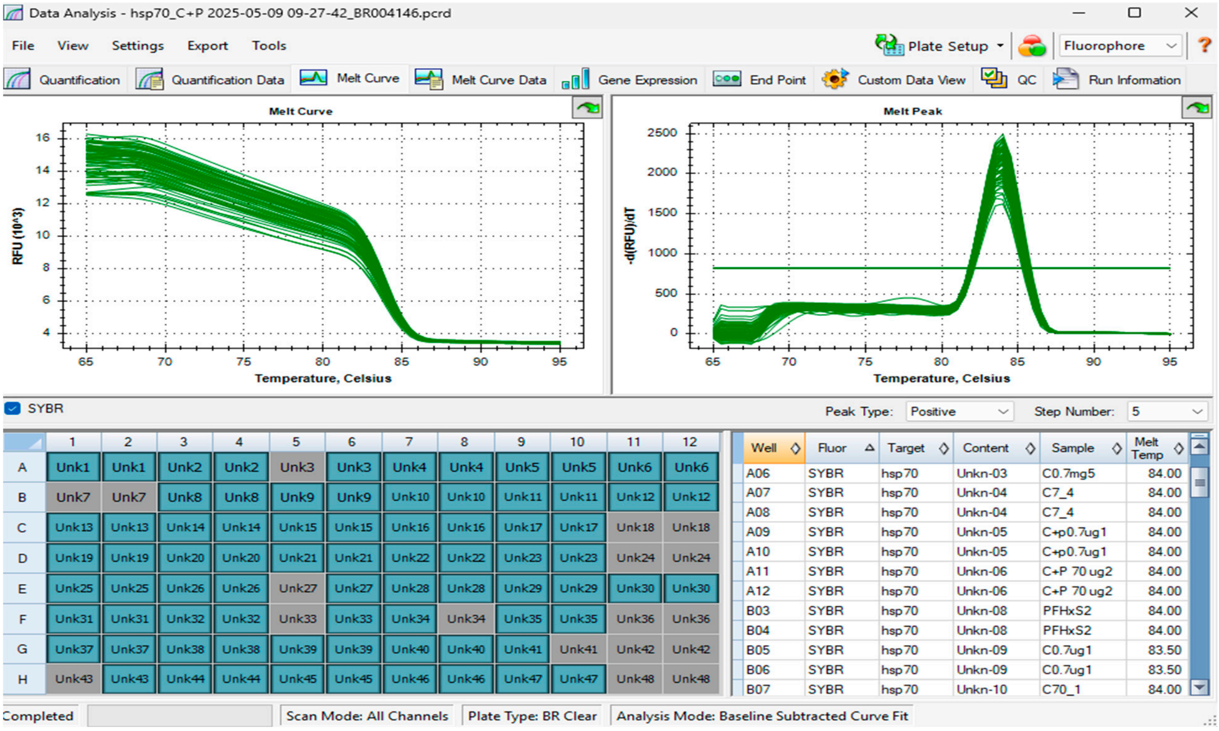
Data Analysis - hsp70_C+P 2025-05-09 09-27-42_BR004146.pcrd
File View Settings Export Tools
Plate Setup
Fluorophore
Quantification Quantification Data Melt Curve Melt Curve Data Gene Expression End Point Custom Data View QC Run Information
Melt Curve
RFU (10^3)
Temperature, Celsius
Melt Peak
-d(RFU)/dT
Temperature, Celsius
SYBR
Peak Type: Positive
Step Number: 5
Well Fluor Target Content Sample Melt Temp
A06 SYBR hsp70 Unkn-03 C0.7mg5 84.00
A07 SYBR hsp70 Unkn-04 C7_4 84.00
A08 SYBR hsp70 Unkn-04 C7_4 84.00
A09 SYBR hsp70 Unkn-05 C+p0.7ug1 84.00
A10 SYBR hsp70 Unkn-05 C+p0.7ug1 84.00
A11 SYBR hsp70 Unkn-06 C+P 70 ug2 84.00
A12 SYBR hsp70 Unkn-06 C+P 70 ug2 84.00
B03 SYBR hsp70 Unkn-08 PFHxS2 84.00
B04 SYBR hsp70 Unkn-08 PFHxS2 84.00
B05 SYBR hsp70 Unkn-09 C0.7ug1 83.50
B06 SYBR hsp70 Unkn-09 C0.7ug1 83.50
B07 SYBR hsp70 Unkn-10 C70_1 84.00
Completed
Scan Mode: All Channels
Plate Type: BR Clear
Analysis Mode: Baseline Subtracted Curve Fit

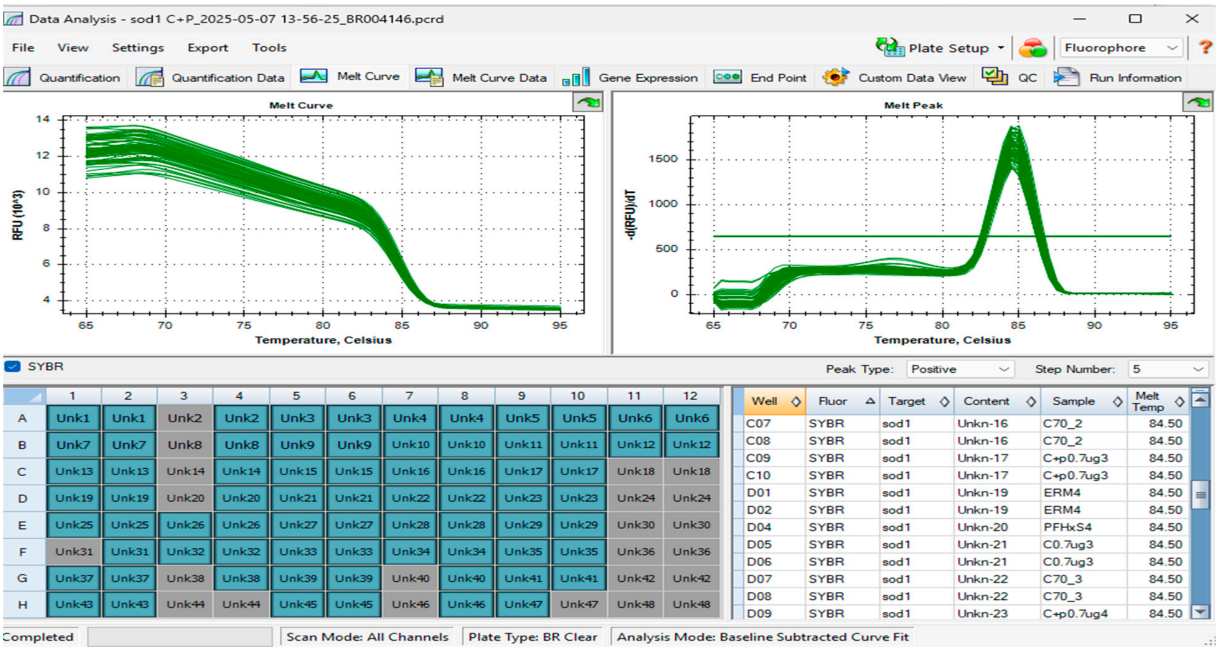
Data Analysis - sod1 C+P_2025-05-07 13-56-25_BR004146.pcrd
File View Settings Export Tools
Plate Setup
Fluorophore
Quantification Quantification Data Melt Curve Melt Curve Data Gene Expression End Point Custom Data View QC Run Information
Melt Curve
RFU (10^3)
Temperature, Celsius
Melt Peak
-d(RFU)/dT
Temperature, Celsius
SYBR
Peak Type: Positive
Step Number: 5
Well Fluor Target Content Sample Melt Temp
C07 SYBR sod1 Unkn-16 C70_2 84.50
C08 SYBR sod1 Unkn-16 C70_2 84.50
C09 SYBR sod1 Unkn-17 C+p0.7ug3 84.50
C10 SYBR sod1 Unkn-17 C+p0.7ug3 84.50
D01 SYBR sod1 Unkn-19 ERM4 84.50
D02 SYBR sod1 Unkn-19 ERM4 84.50
D04 SYBR sod1 Unkn-20 PFHxS4 84.50
D05 SYBR sod1 Unkn-21 C0.7ug3 84.50
D06 SYBR sod1 Unkn-21 C0.7ug3 84.50
D07 SYBR sod1 Unkn-22 C70_3 84.50
D08 SYBR sod1 Unkn-22 C70_3 84.50
D09 SYBR sod1 Unkn-23 C+p0.7ug4 84.50
Completed
Scan Mode: All Channels
Plate Type: BR Clear
Analysis Mode: Baseline Subtracted Curve Fit

Genes related to neurotoxicity

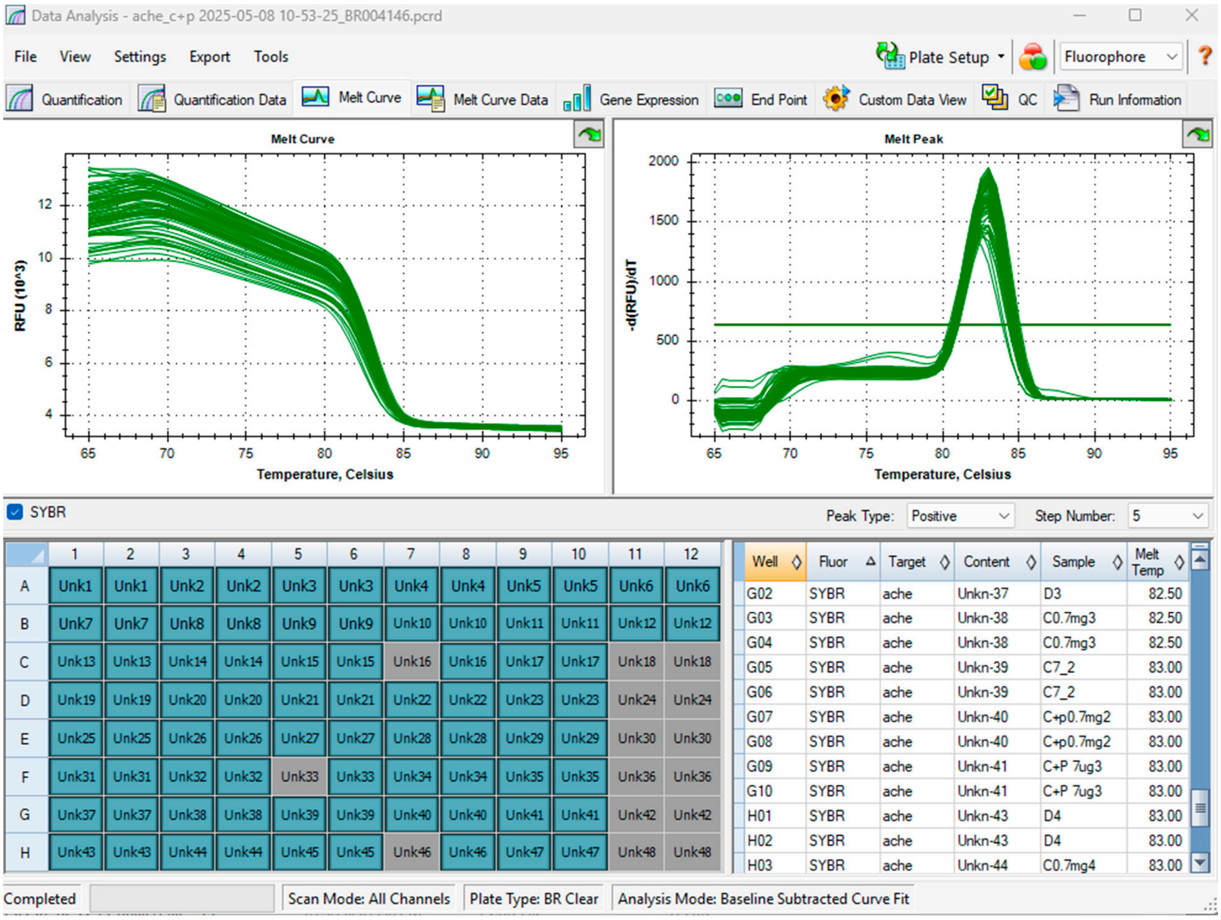

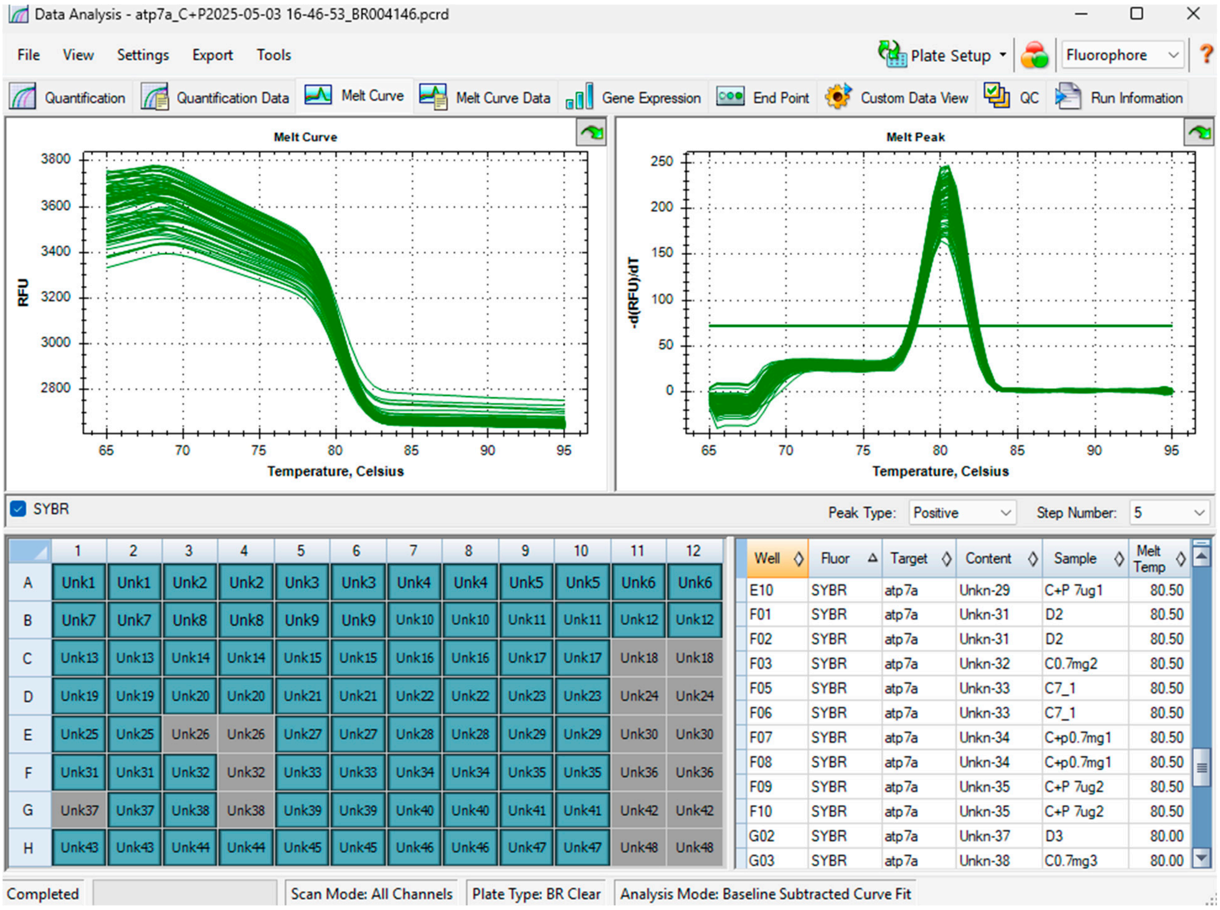
Data Analysis - atp7a_C+P2025-05-03 16-46-53_BR004146.pcrd
File View Settings Export Tools
Plate Setup
Fluorophore
Quantification
Quantification Data
Melt Curve
Melt Curve Data
Gene Expression
End Point
Custom Data View
QC
Run Information
Melt Curve
RFU
Temperature, Celsius
Melt Peak
-d(RFU)/dT
Temperature, Celsius
SYBR
Peak Type: Positive
Step Number: 5
Well Fluor Target Content Sample Melt Temp
E10 SYBR atp7a Unkn-29 C+P 7ug1 80.50
F01 SYBR atp7a Unkn-31 D2 80.50
F02 SYBR atp7a Unkn-31 D2 80.50
F03 SYBR atp7a Unkn-32 C0.7mg2 80.50
F05 SYBR atp7a Unkn-33 C7_1 80.50
F06 SYBR atp7a Unkn-33 C7_1 80.50
F07 SYBR atp7a Unkn-34 C+p0.7mg1 80.50
F08 SYBR atp7a Unkn-34 C+p0.7mg1 80.50
F09 SYBR atp7a Unkn-35 C+P 7ug2 80.50
F10 SYBR atp7a Unkn-35 C+P 7ug2 80.50
G02 SYBR atp7a Unkn-37 D3 80.00
G03 SYBR atp7a Unkn-38 C0.7mg3 80.00
Completed
Scan Mode: All Channels
Plate Type: BR Clear
Analysis Mode: Baseline Subtracted Curve Fit

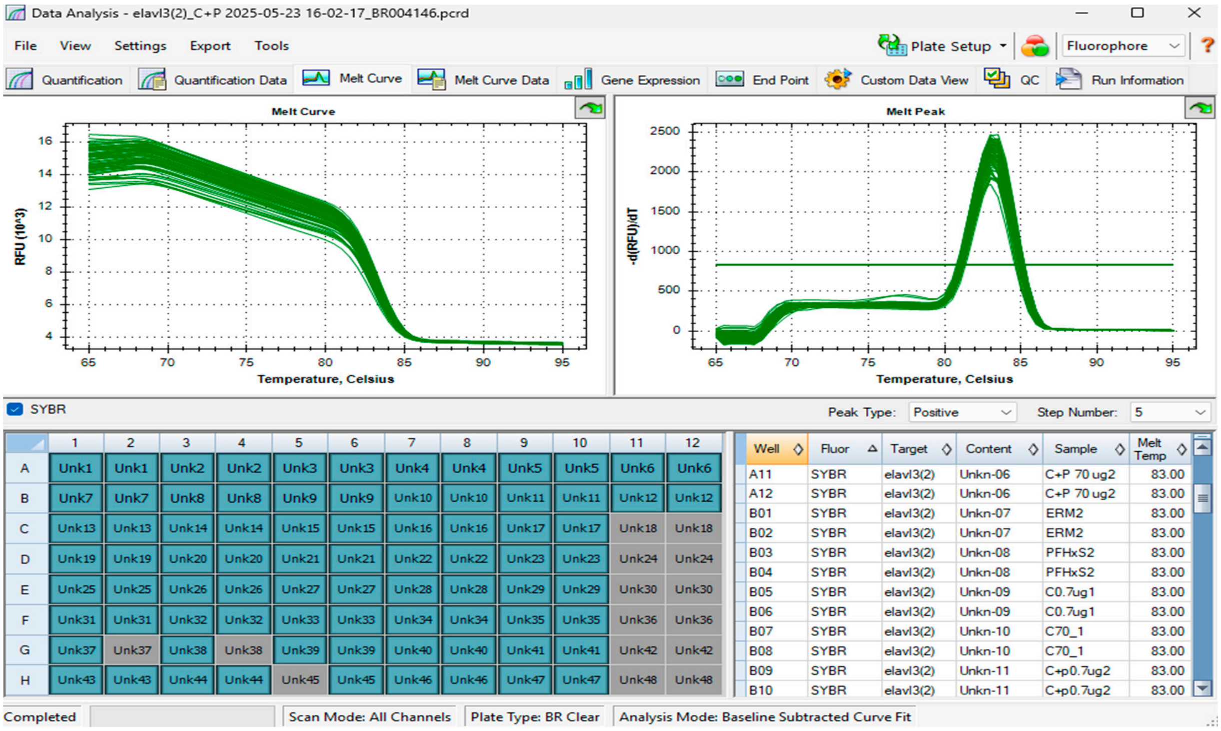

Data Analysis - elavl3(2)_C+P 2025-05-23 16-02-17_BR004146.pcrd
File View Settings Export Tools
Plate Setup
Fluorophore
Quantification Quantification Data Melt Curve Melt Curve Data Gene Expression End Point Custom Data View QC Run Information
Melt Curve
RFU (10^3)
Temperature, Celsius
Melt Peak
-d(RFU)/dT
Temperature, Celsius
SYBR
Peak Type: Positive
Step Number: 5
Well Fluor Target Content Sample Melt Temp
A11 SYBR elavl3(2) Unkn-06 C+P 70 ug2 83.00
A12 SYBR elavl3(2) Unkn-06 C+P 70 ug2 83.00
B01 SYBR elavl3(2) Unkn-07 ERM2 83.00
B02 SYBR elavl3(2) Unkn-07 ERM2 83.00
B03 SYBR elavl3(2) Unkn-08 PFHxS2 83.00
B04 SYBR elavl3(2) Unkn-08 PFHxS2 83.00
B05 SYBR elavl3(2) Unkn-09 C0.7ug1 83.00
B06 SYBR elavl3(2) Unkn-09 C0.7ug1 83.00
B07 SYBR elavl3(2) Unkn-10 C70_1 83.00
B08 SYBR elavl3(2) Unkn-10 C70_1 83.00
B09 SYBR elavl3(2) Unkn-11 C+p0.7ug2 83.00
B10 SYBR elavl3(2) Unkn-11 C+p0.7ug2 83.00
Completed
Scan Mode: All Channels
Plate Type: BR Clear
Analysis Mode: Baseline Subtracted Curve Fit

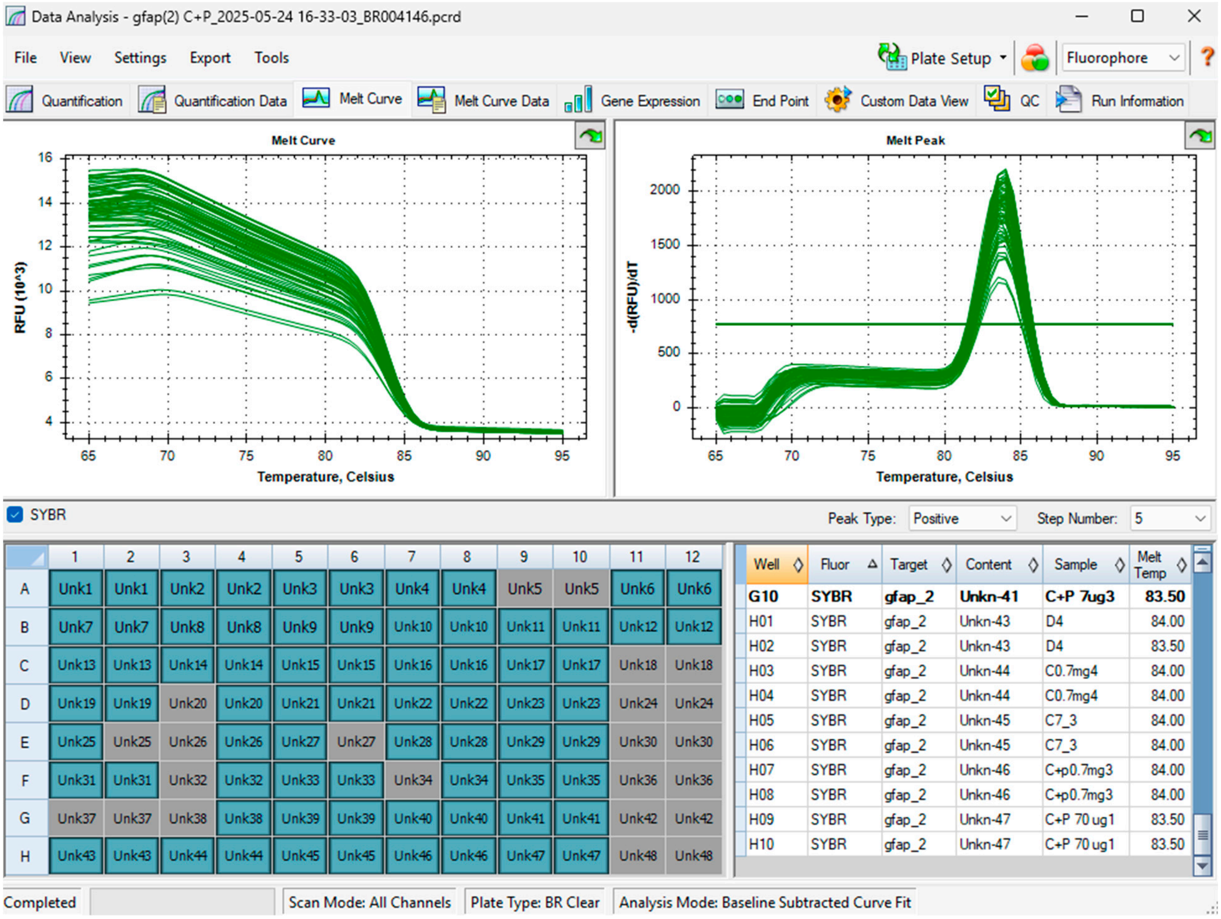

Data Analysis - gfap(2) C+P_2025-05-24 16-33-03_BR004146.pcrd
File View Settings Export Tools
Plate Setup
Fluorophore
Quantification Quantification Data Melt Curve Melt Curve Data Gene Expression End Point Custom Data View QC Run Information
Melt Curve
RFU (10^3)
Temperature, Celsius
Melt Peak
-d(RFU)/dT
Temperature, Celsius
SYBR
Peak Type: Positive
Step Number: 5
Well Fluor Target Content Sample Melt Temp
G10 SYBR gfap_2 Unkn-41 C+P 7ug3 83.50
H01 SYBR gfap_2 Unkn-43 D4 84.00
H02 SYBR gfap_2 Unkn-43 D4 83.50
H03 SYBR gfap_2 Unkn-44 C0.7mg4 84.00
H04 SYBR gfap_2 Unkn-44 C0.7mg4 84.00
H05 SYBR gfap_2 Unkn-45 C7_3 84.00
H06 SYBR gfap_2 Unkn-45 C7_3 84.00
H07 SYBR gfap_2 Unkn-46 C+p0.7mg3 84.00
H08 SYBR gfap_2 Unkn-46 C+p0.7mg3 84.00
H09 SYBR gfap_2 Unkn-47 C+P 70 ug1 83.50
H10 SYBR gfap_2 Unkn-47 C+P 70 ug1 83.50
Completed
Scan Mode: All Channels
Plate Type: BR Clear
Analysis Mode: Baseline Subtracted Curve Fit

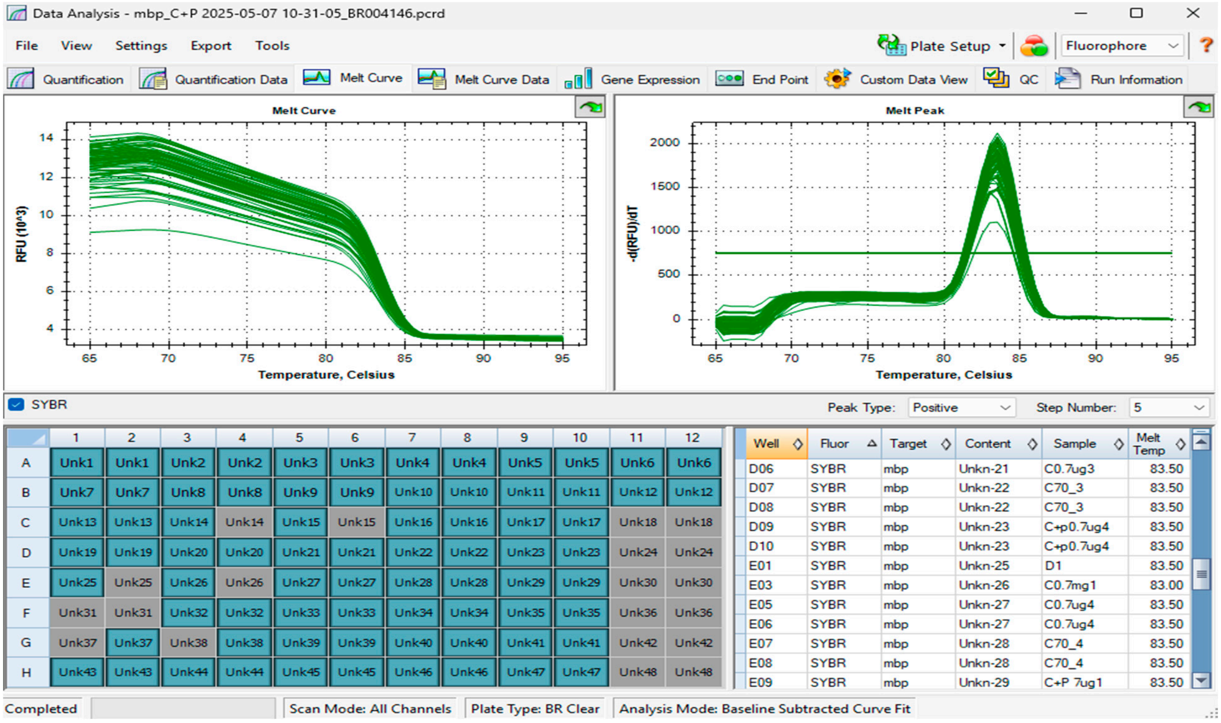

Well	Fluor	Target	Content	Sample	Melt Temp
D06	SYBR	mbp	Unkn-21	C0.7ug3	83.50
D07	SYBR	mbp	Unkn-22	C70_3	83.50
D08	SYBR	mbp	Unkn-22	C70_3	83.50
D09	SYBR	mbp	Unkn-23	C+p0.7ug4	83.50
D10	SYBR	mbp	Unkn-23	C+p0.7ug4	83.50
E01	SYBR	mbp	Unkn-25	D1	83.50
E03	SYBR	mbp	Unkn-26	C0.7mg1	83.00
E05	SYBR	mbp	Unkn-27	C0.7ug4	83.50
E06	SYBR	mbp	Unkn-27	C0.7ug4	83.50
E07	SYBR	mbp	Unkn-28	C70_4	83.50
E08	SYBR	mbp	Unkn-28	C70_4	83.50
E09	SYBR	mbp	Unkn-29	C+P 7ug1	83.50

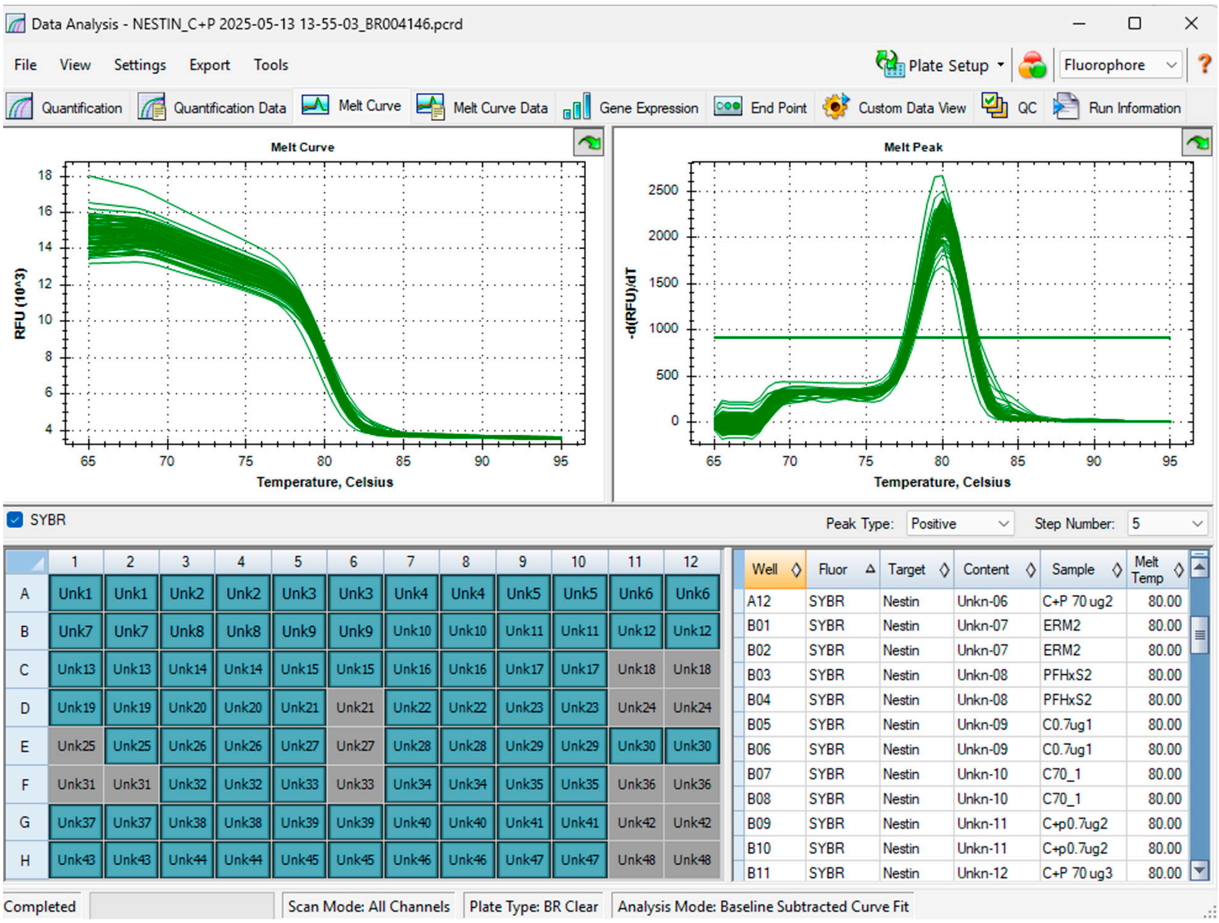
Data Analysis - NESTIN_C+P 2025-05-13 13-55-03_BR004146.pcrd
File
View
Settings
Export
Tools
Plate Setup
Fluorophore
Quantification
Quantification Data
Melt Curve
Melt Curve Data
Gene Expression
End Point
Custom Data View
QC
Run Information
Melt Curve
RFU (10^3)
Temperature, Celsius
Melt Peak
-d(RFU)/dT
Temperature, Celsius
SYBR
Peak Type: Positive
Step Number: 5
Well Fluor Target Content Sample Melt Temp
A12 SYBR Nestin Unkn-06 C+P 70 ug2 80.00
B01 SYBR Nestin Unkn-07 ERM2 80.00
B02 SYBR Nestin Unkn-07 ERM2 80.00
B03 SYBR Nestin Unkn-08 PFHxS2 80.00
B04 SYBR Nestin Unkn-08 PFHxS2 80.00
B05 SYBR Nestin Unkn-09 C0.7ug1 80.00
B06 SYBR Nestin Unkn-09 C0.7ug1 80.00
B07 SYBR Nestin Unkn-10 C70_1 80.00
B08 SYBR Nestin Unkn-10 C70_1 80.00
B09 SYBR Nestin Unkn-11 C+p0.7ug2 80.00
B10 SYBR Nestin Unkn-11 C+p0.7ug2 80.00
B11 SYBR Nestin Unkn-12 C+P 70 ug3 80.00
Completed
Scan Mode: All Channels
Plate Type: BR Clear
Analysis Mode: Baseline Subtracted Curve Fit

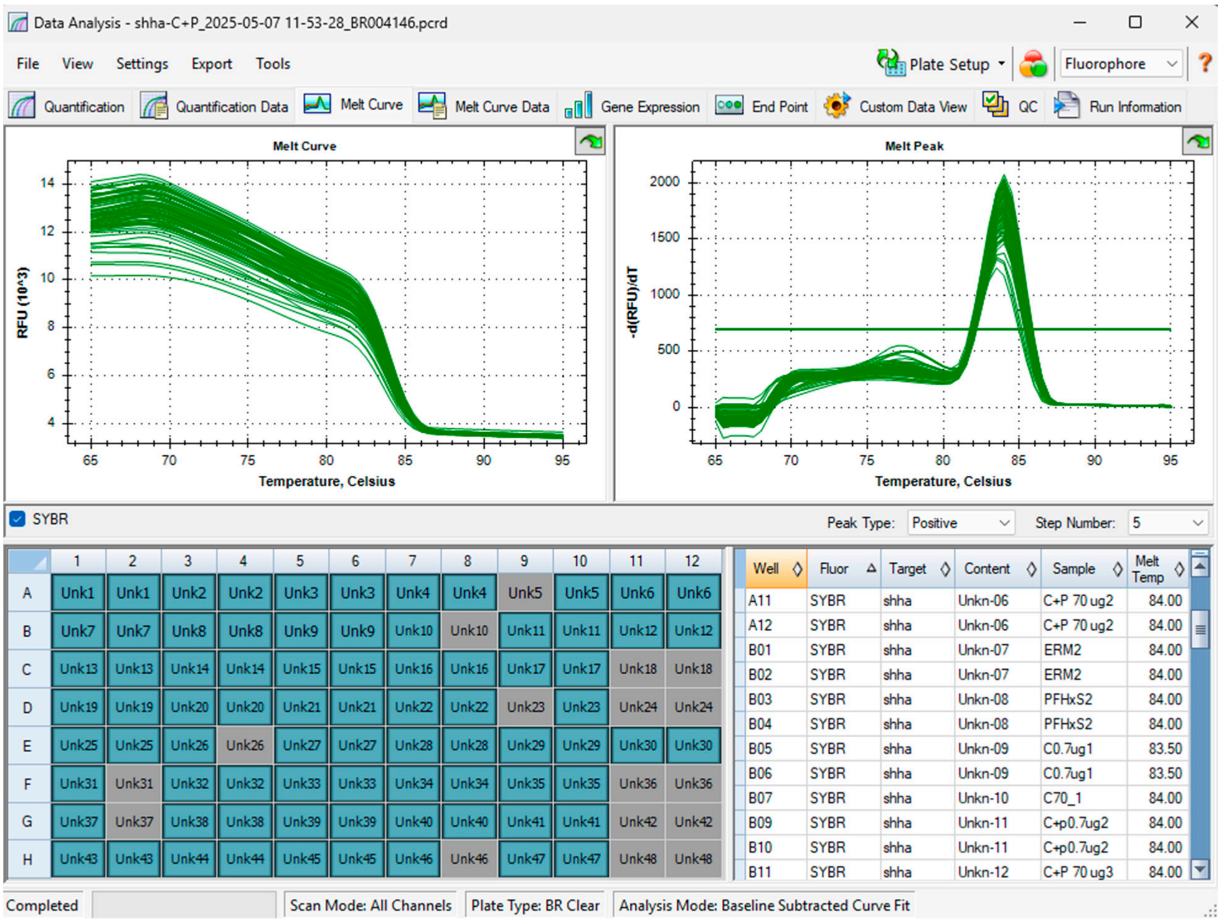

Data Analysis - shha-C+P_2025-05-07 11-53-28_BR004146.pcrd
File View Settings Export Tools
Plate Setup
Fluorophore
Quantification
Quantification Data
Melt Curve
Melt Curve Data
Gene Expression
End Point
Custom Data View
QC
Run Information
Melt Curve
RFU (10^3)
Temperature, Celsius
Melt Peak
-d(RFU)/dT
Temperature, Celsius
SYBR
Peak Type: Positive
Step Number: 5
1 2 3 4 5 6 7 8 9 10 11 12
A Unk1 Unk1 Unk2 Unk2 Unk3 Unk3 Unk4 Unk4 Unk5 Unk5 Unk6 Unk6
B Unk7 Unk7 Unk8 Unk8 Unk9 Unk9 Unk10 Unk10 Unk11 Unk11 Unk12 Unk12
C Unk13 Unk13 Unk14 Unk14 Unk15 Unk15 Unk16 Unk16 Unk17 Unk17 Unk18 Unk18
D Unk19 Unk19 Unk20 Unk20 Unk21 Unk21 Unk22 Unk22 Unk23 Unk23 Unk24 Unk24
E Unk25 Unk25 Unk26 Unk26 Unk27 Unk27 Unk28 Unk28 Unk29 Unk29 Unk30 Unk30
F Unk31 Unk31 Unk32 Unk32 Unk33 Unk33 Unk34 Unk34 Unk35 Unk35 Unk36 Unk36
G Unk37 Unk37 Unk38 Unk38 Unk39 Unk39 Unk40 Unk40 Unk41 Unk41 Unk42 Unk42
H Unk43 Unk43 Unk44 Unk44 Unk45 Unk45 Unk46 Unk46 Unk47 Unk47 Unk48 Unk48
Well Fluor Target Content Sample Melt Temp
A11 SYBR shha Unkn-06 C+P 70 ug2 84.00
A12 SYBR shha Unkn-06 C+P 70 ug2 84.00
B01 SYBR shha Unkn-07 ERM2 84.00
B02 SYBR shha Unkn-07 ERM2 84.00
B03 SYBR shha Unkn-08 PFHxS2 84.00
B04 SYBR shha Unkn-08 PFHxS2 84.00
B05 SYBR shha Unkn-09 C0.7ug1 83.50
B06 SYBR shha Unkn-09 C0.7ug1 83.50
B07 SYBR shha Unkn-10 C70_1 84.00
B09 SYBR shha Unkn-11 C+p0.7ug2 84.00
B10 SYBR shha Unkn-11 C+p0.7ug2 84.00
B11 SYBR shha Unkn-12 C+P 70 ug3 84.00
Completed
Scan Mode: All Channels
Plate Type: BR Clear
Analysis Mode: Baseline Subtracted Curve Fit

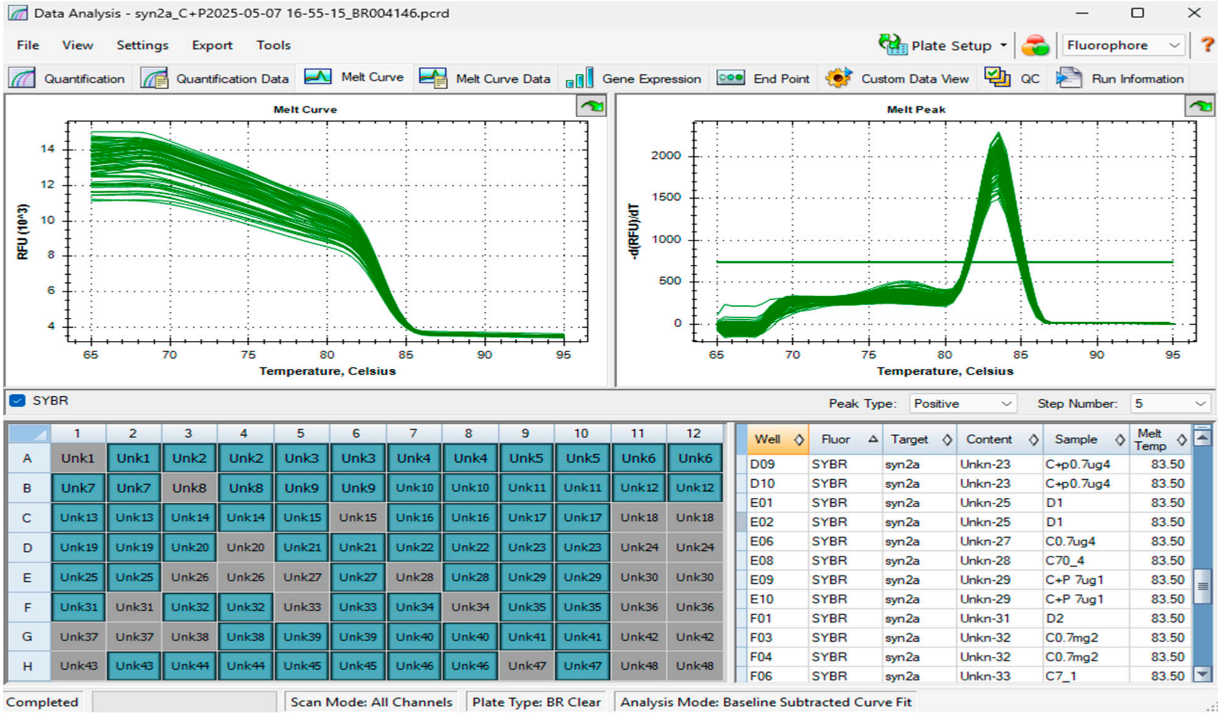
Data Analysis - syn2a_C+P2025-05-07 16-55-15_BR004146.pcrd
File View Settings Export Tools
Plate Setup
Fluorophore
Quantification
Quantification Data
Melt Curve
Melt Curve Data
Gene Expression
End Point
Custom Data View
QC
Run Information
Melt Curve
RFU (10^3)
Temperature, Celsius
Melt Peak
-d(RFU)/dT
Temperature, Celsius
SYBR
Peak Type: Positive
Step Number: 5
Well Fluor Target Content Sample Melt Temp
D09 SYBR syn2a Unkn-23 C+p0.7ug4 83.50
D10 SYBR syn2a Unkn-23 C+p0.7ug4 83.50
E01 SYBR syn2a Unkn-25 D1 83.50
E02 SYBR syn2a Unkn-25 D1 83.50
E06 SYBR syn2a Unkn-27 C0.7ug4 83.50
E08 SYBR syn2a Unkn-28 C70_4 83.50
E09 SYBR syn2a Unkn-29 C+P 7ug1 83.50
E10 SYBR syn2a Unkn-29 C+P 7ug1 83.50
F01 SYBR syn2a Unkn-31 D2 83.50
F03 SYBR syn2a Unkn-32 C0.7mg2 83.50
F04 SYBR syn2a Unkn-32 C0.7mg2 83.50
F06 SYBR syn2a Unkn-33 C7_1 83.50
Completed
Scan Mode: All Channels
Plate Type: BR Clear
Analysis Mode: Baseline Subtracted Curve Fit

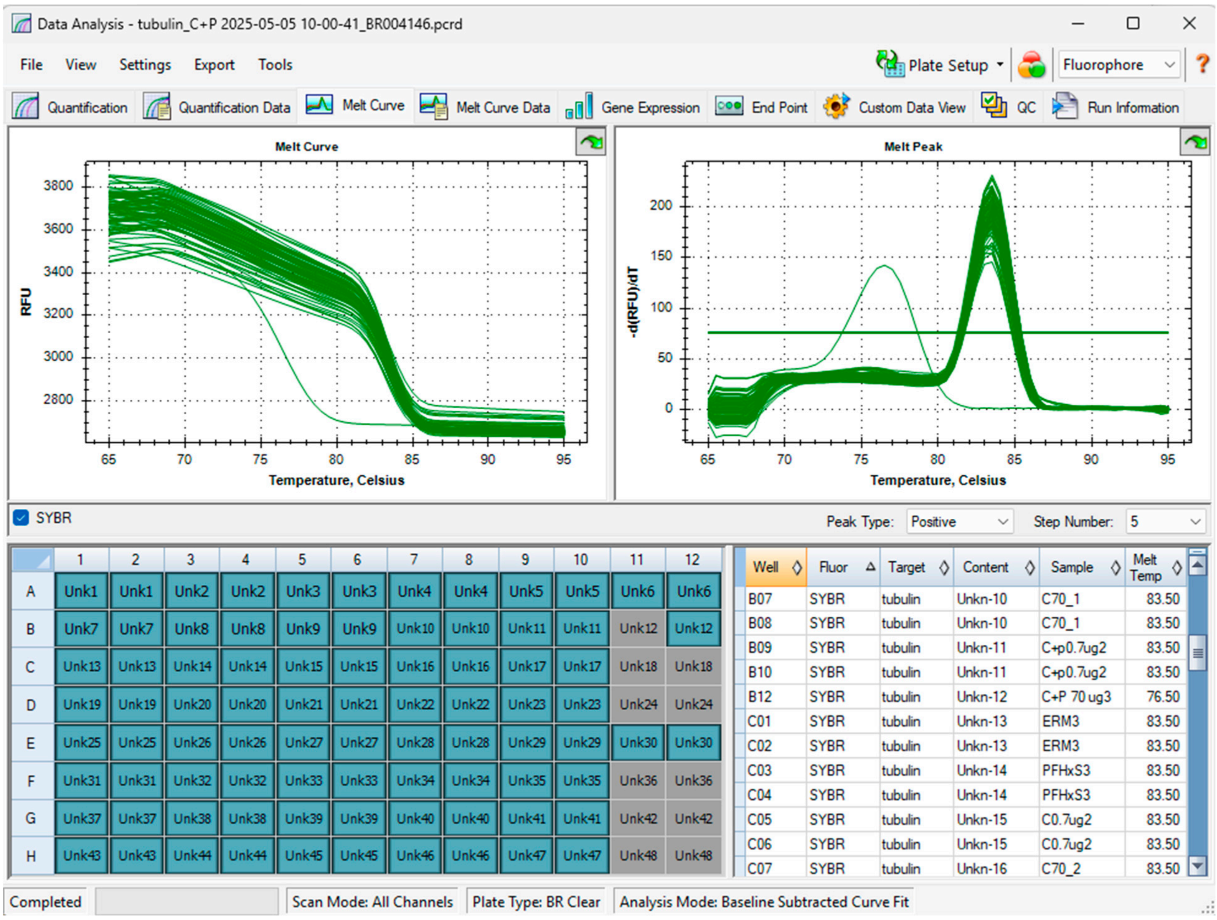

Data Analysis - tubulin_C+P 2025-05-05 10-00-41_BR004146.pcrd
File
View
Settings
Export
Tools
Plate Setup
Fluorophore
Quantification
Quantification Data
Melt Curve
Melt Curve Data
Gene Expression
End Point
Custom Data View
QC
Run Information
Melt Curve
RFU
Temperature, Celsius
Melt Peak
-d(RFU)/dT
Temperature, Celsius
SYBR
Peak Type:
Positive
Step Number:
5
Well
Fluor
Target
Content
Sample
Melt Temp
B07 SYBR tubulin Unkn-10 C70_1 83.50
B08 SYBR tubulin Unkn-10 C70_1 83.50
B09 SYBR tubulin Unkn-11 C+p0.7ug2 83.50
B10 SYBR tubulin Unkn-11 C+p0.7ug2 83.50
B12 SYBR tubulin Unkn-12 C+P 70 ug3 76.50
C01 SYBR tubulin Unkn-13 ERM3 83.50
C02 SYBR tubulin Unkn-13 ERM3 83.50
C03 SYBR tubulin Unkn-14 PFHxS3 83.50
C04 SYBR tubulin Unkn-14 PFHxS3 83.50
C05 SYBR tubulin Unkn-15 C0.7ug2 83.50
C06 SYBR tubulin Unkn-15 C0.7ug2 83.50
C07 SYBR tubulin Unkn-16 C70_2 83.50
Completed
Scan Mode: All Channels
Plate Type: BR Clear
Analysis Mode: Baseline Subtracted Curve Fit
