# Supplementary material for: Neurobehavioral and Molecular Alterations Following Single and Combined Exposure to Chlorpyrifos and PFHxS in Developing Zebrafish (Danio rerio)
Source: Toxics. 2026 Jun 27;14(7):566. doi: 10.3390/toxics14070566 (PMC13418585; doi:10.3390/toxics14070566)
Supplement: Supplementary file 1 [file toxics-14-00566-s001.zip › toxics-4348990 Supplemental File S2 data_Valle et al_R1.pdf]

Neurobehavioral and molecular alterations following single and combined exposure to chlorpyrifos and PFHxS in developing zebrafish (*Danio rerio*)

Eliana Maira Agostini Valle[1,2], Amany Sultan[1,3], Michelle Puerta[1], Romanna Shams[1,4], Jack Reites[1], Isaac Konig[1,5], and Christopher J. Martyniuk[1, 6*]

[1] Center for Environmental and Human Toxicology, Department of Physiological Sciences, College of Veterinary Medicine, University of Florida, Gainesville, Florida, 32611, USA

[2] Universidade Federal de São Paulo – Instituto de Ciências Ambientais, Químicas e Farmacêuticas – Campus Diadema – Brasil

[3] Animal Health Research Institute, Agriculture Research Center (ARC), Egypt
[4] University of Veterinary and Animal Sciences, Lahore, Pakistan
[5] Department of Biochemistry, Federal University of Rio Grande do Sul (UFRGS), Porto Alegre, Rio Grande do Sul, Brazil

[6] UF Genetics Institute, Interdisciplinary Program in Biomedical Sciences& Neuroscience, University of Florida, USA

*Correspondence:
Chris Martyniuk, email: cmartyn@ufl.edu
ORCID: 0000-0003-0921-4796

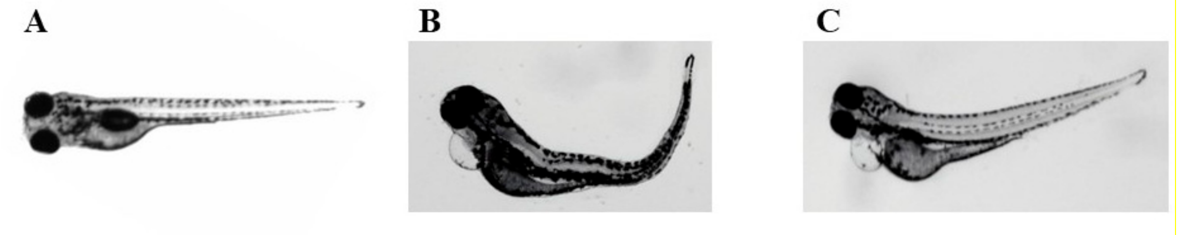

Figure S1. Selected photomicrographs of morphological deformities observed in zebrafish larvae at 5 days after being exposed to A) ERM, B) 700 µg/L of CPF, and C) 700 µg/L CPF + 10 µg/L of PFHxS. Deformities observed (B and C) included pericardial edema (PE), yolk sac edema (YSE), and spinal lordosis (SL).

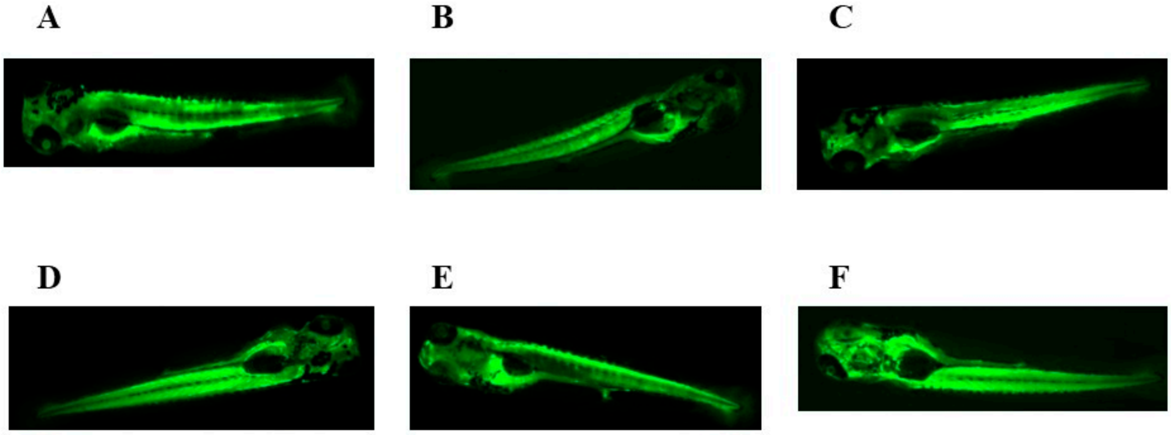

Figure S2. Selected photomicrographs of acridine orange staining in zebrafish larvae at 5 days after being exposed to A) ERM, B) DMSO (0.1%), C) 10 µg/L PFHxS, D) 0.7 µg/L CPF, E) 700 µg/L CPF, F) 700 µg/L and + 10 µg/L of PFHxS. No differences were observed among treatment groups.
