# Supplementary material for: Neurobehavioral and Molecular Alterations Following Single and Combined Exposure to Chlorpyrifos and PFHxS in Developing Zebrafish (Danio rerio)
Source: Toxics. 2026 Jun 27;14(7):566. doi: 10.3390/toxics14070566 (PMC13418585; doi:10.3390/toxics14070566)
Supplement: Supplementary file 1 [file toxics-14-00566-s001.zip › toxics-4348990 Supplemental File S3 methods_Valle et al., 2026_R1.pdf]

Neurobehavioral and molecular alterations following single and combined exposure to chlorpyrifos and PFHxS in developing zebrafish (*Danio rerio*)

Eliana Maira Agostini Valle[1,2], Amany Sultan[1,3], Michelle Puerta[1], Romanna Shams[1,4], Jack Reites[1], Isaac Konig[1,5], and Christopher J. Martyniuk[1, 6*]

[1] Center for Environmental and Human Toxicology, Department of Physiological Sciences, College of Veterinary Medicine, University of Florida, Gainesville, Florida, 32611, USA

[2] Universidade Federal de São Paulo – Instituto de Ciências Ambientais, Químicas e Farmacêuticas – Campus Diadema – Brasil

[3] Animal Health Research Institute, Agriculture Research Center (ARC), Egypt
[4] University of Veterinary and Animal Sciences, Lahore, Pakistan
[5] Department of Biochemistry, Federal University of Rio Grande do Sul (UFRGS), Porto Alegre, Rio Grande do Sul, Brazil

[6] UF Genetics Institute, Interdisciplinary Program in Biomedical Sciences& Neuroscience, University of Florida, USA

*Correspondence:
Chris Martyniuk, email: cmartyn@ufl.edu
ORCID: 0000-0003-0921-4796

SUPPLEMENTARY METHODS

RNA extraction and purification

Total RNA was extracted using Trizol® reagent, employing exclusively RNase- and DNase-free materials to prevent RNA degradation. The larvae were kept frozen throughout the initial handling process. For extraction, 750 µL of Trizol® was added to each tube containing the samples, which were then homogenized using a tissue homogenizer. Homogenization was performed in short 3–5 s bursts, totaling approximately 30 s, while the samples were maintained on ice to minimize heating and preserve RNA integrity.

Following homogenization, the samples were incubated at room temperature for 5 min. Subsequently, 150 µL of chloroform was added to each tube, followed by vigorous shaking for 15 s and incubation at room temperature for 2 min. The samples were then centrifuged at 12,000 × g for 15 min at 4 °C to achieve phase separation. The upper aqueous phase containing the RNA was carefully transferred to clean tubes, avoiding contamination from the interphase and organic phase.

RNA precipitation was carried out by adding 750 µL of isopropanol to the aqueous phase, followed by mixing by inversion and incubation at room temperature for 10 min. The samples were subsequently centrifuged at 12,000 × g for 10 min at 4 °C to pellet the RNA. The supernatant was discarded, and the RNA pellet was washed with 1 mL of 75% ethanol prepared with DEPC-treated water. After vortexing for 30 s, the samples were centrifuged at 12,000 × g for 5 min at 4 °C. The supernatant was removed, and the pellets were air-dried at room temperature for approximately 5 min. Finally, the RNA pellet was resuspended in 15 µL of RNase- and DNase-free water.

The RNA samples were further purified by adding 80 µL of nuclease-free water. Subsequently, 350 µL of RLT buffer was added and mixed thoroughly. Then, 250 µL of absolute ethanol (100%) was carefully incorporated into the solution. The mixture was transferred to an RNeasy Mini Spin Column fitted into a 2 mL collection tube and centrifuged at 10,000 × g for 15 s. The flow-through was discarded, and 500 µL of RPE buffer was added to the spin column, followed by centrifugation at 10,000 × g for 15 s. Finally, 25 µL of nuclease-free water was added directly onto the RNeasy spin column membrane, which was placed into a 1.5 mL

microcentrifuge tube. After centrifugation, the purified RNA was collected in the microcentrifuge tube and used for subsequent analyses.

Supplemental Table S1: Primer sequences used for qPCR.

Gene Symbol	Gene Name	Forward Primer (5' to 3')	Reverse Primer (5' to 3')	Reference
**Housekeeping**				
β-actin	Beta-actin	CGAGCAGGAGATGGGAACC	CAACGGAAACGCTCATTGC	Wang et al., 2018
rps18	Ribosomal subunit 18	TCGCTAGTTGGCATCGTTTATG	CGGAGGTTCGAAGACGATCA	Wang et al., 2018
rpl13a	Ribosomal Protein L13a	AGCTCAAGATGGCAACACAG	AAGTTCTTCTCGTCCTCC	Zucchi et al., 2011
**Oxidative stress**				
cat	Catalase	CTCCTGATGTGGCCCGATAC	TCAGATGCCCGGCCATATTC	Sarkar et al., 2014
hsp70	Heat shock protein 70			
sod1	Superoxide dismutase 1	CGTCTATTTCAATCAAGAGGGTG	GATGCAGCCGTTTGTGTTGTC	Lin et al., 2009
**Neurotoxicity**				
ache	acetylcholinesterase	GCTAATGAGCAAAAGCATGTGGGC	TATCTGTGATGTTAAGCAGACGAGGCA	NM_131846.2
atp7a	ATPase copper transporting alpha			
elavl3	ELAV-like neuron-specific RNA binding protein 3	AGACAAGATCACAGGCCAGAGCTT	TGGTCTGCAGTTTGAGACCGTTGA	NM_131449.1<br>Yang et al., 2023
gfap	glial fibrillary acidic protein	GGATGCAGCCAATCGTAAT	TTCCAGGTCACAGGTCAG	NM_131373.2
mbp	myelin basic protein	AATCAGCAGGTTCTTCGGAGGAGA	AAGAAATGCACGACAGGGTTGACG	BC115202.1<br>Yang et al., 2023
nestin	nestin intermediate filament protein	ATGCTGGAGAAACATGCCATGCAG	AGGGTGTTTACTTGGGCCTGAAGA	XM_001919887<br>Jiang et al., 2018
shha	sonic hedgehog a	AGACCGAGACTCCACGACGC	TGCAGTCACTGGTGCGAACG	Guo et al., 2023
syn2a	synapsin IIa	GTACCATGCCAGCATTTC	TGGTTCTCCACTTTCACCTT	Guo et al., 2023
α1-tubulin	tubulin alpha 1	AATCACCAATGCTTGCTTCGAGCC	TTCACGTCTTTGGGTACCACGTCA	NM_194388.2

References:

Guo, Y., Fu, Y., & Sun, W. (2023). 50 Hz Magnetic Field Exposure Inhibited Spontaneous Movement of Zebrafish Larvae through ROS-Mediated syn2a Expression. International journal of molecular sciences, 24(8), 7576. https://doi.org/10.3390/ijms24087576

Jiang, F., Liu, J., Zeng, X., Yu, L., Liu, C. and Wang, J., 2018. Tris (2-butoxyethyl) phosphate affects motor behavior and axonal growth in zebrafish (*Danio rerio*) larvae. Aquatic Toxicology, 198, pp.215-223.

Lin, C.T., Tseng, W.C., Hsiao, N.W., Chang, H.H., Ken, C.F., 2009. Characterization, molecular modelling and developmental expression of zebrafish manganese superoxide dismutase. Fish Shellfish Immunol. 27, 318-324.

Sarkar, S., Mukherjee, S., Chattopadhyay, A. and Bhattacharya, S., 2014. Low dose of arsenic trioxide triggers oxidative stress in zebrafish brain: expression of antioxidant genes. Ecotoxicology and environmental safety, 107, pp.1-8.

Wang, X.H., Souders 2nd, C.L., Zhao, Y.H., Martyniuk, C.J. 2018. Paraquat affects mitochondrial bioenergetics, dopamine system expression, and locomotor activity in zebrafish (*Danio rerio*). Chemosphere. 191, 106-117.

Zucchi, S., Blüthgen, N., Ieronimo, A., Fent, K., 2011. The UV-absorber benzophenone-4 alters transcripts of genes involved in hormonal pathways in zebrafish (*Danio rerio*) eleuthero-embryos and adult males. Toxicol. Appl. Pharmacol. 250, 137-146.

Yang Q, Deng P, Xing D, Liu H, Shi F, Hu L, Zou X, Nie H, Zuo J, Zhuang Z, Pan M, Chen J, Li G. Developmental Neurotoxicity of Difenoconazole in Zebrafish Embryos. Toxics. 2023 Apr 8;11(4):353. doi: 10.3390/toxics11040353. PMID: 37112580; PMCID: PMC10142703.
